# Low-intensity transcranial ultrasound neuromodulation promotes neuronal regeneration: A new hope for noninvasive treatment of neurodegenerative diseases

**DOI:** 10.4103/NRR.NRR-D-25-00113

**Published:** 2025-08-13

**Authors:** Shu Xia, Chen He, Yunfei Li, Hao Li, Bo Wang, Long Xu, Xudong Zhao

**Affiliations:** 1 Kangda College, Nanjing Medical University, Lianyungang, Jiangsu Province, China; 2 Department of Neurosurgery, Beijing Tiantan Hospital, Capital Medical University, Beijing, China; 3 Institute of Artificial Intelligence, Hefei Comprehensive National Science Center, Hefei, Anhui Province, China; 4 Institute of Advanced Technology, University of Science and Technology of China, Hefei, Anhui Province, China; 5 State Key Laboratory of Brain and Cognitive Science, Institute of Biophysics, Chinese Academy of Sciences, Beijing, China; 6 CAS Center for Excellence in Brain Science and Intelligence Technology, Beijing, China; 7 University of Chinese Academy of Sciences, Beijing, China; 8 China National Clinical Research Center for Neurological Diseases, Beijing, China

**Keywords:** Alzheimer’s disease, frontotemporal dementia, low-intensity transcranial ultrasound, multiple sclerosis, multiple system atrophy, neurodegenerative diseases, neuromodulation, neuronal regeneration, Parkinson’s disease, transcranial ultrasound stimulation

## Abstract

Neurodegenerative diseases, which are characterized by progressive neuronal loss and the lack of disease-modifying therapies, are becoming a major global health challenge. The existing neuromodulation techniques, such as deep brain stimulation and transcranial magnetic stimulation, show limitations such as invasiveness, restricted cortical targeting, and irreversible tissue effects. In this context, low-intensity transcranial ultrasound has emerged as a promising noninvasive alternative that can penetrate deep into the brain and modulate neuroplasticity. This review comprehensively assesses the therapeutic mechanisms, efficacy, and translational potential of low-intensity transcranial ultrasound in treating neurodegenerative diseases, with emphasis on its role in promoting neuronal regeneration, modulating neuroinflammation, and enhancing functional recovery. We summarize the findings of previous studies and systematically illustrate the potential of low-intensity transcranial ultrasound in regulating cell death mechanisms, enhancing neural repair and regeneration, and alleviating symptoms associated with neurodegenerative diseases. Preclinical findings indicate that low-intensity transcranial ultrasound can enhance the release of neurotrophic factors (e.g., brain-derived neurotrophic factor), promote autophagy to clear protein aggregates, modulate microglial activation, and temporarily open the blood–brain barrier to facilitate targeted drug delivery. Existing clinical trial data show that low-intensity transcranial ultrasound can reduce amyloid-β plaques, improve motor and cognitive deficits, and promote remyelination in various disease models. Early clinical trials suggest that low-intensity transcranial ultrasound may enhance cognitive scores in Alzheimer’s disease and alleviate motor symptoms in Parkinson’s disease, all while demonstrating a favorable safety profile. Past studies support the notion that by integrating safety, precision, and reversibility, low-intensity transcranial ultrasound can transform the treatment landscape for neurodegenerative disease. However, more advancements are necessary for future clinical application of low-intensity transcranial ultrasound, including optimizing parameters such as frequency, intensity, and duty cycle; considering individual anatomical differences; and confirming long-term efficacy. We believe establishing standardized protocols, conducting larger trials, and investigating the underlying mechanisms to clarify dose-response relationships and refine personalized application strategies are essential in this regard. Future research should focus on translating preclinical findings into clinical practice, addressing technical challenges, and exploring combination therapies with pharmacological or gene interventions.

## Introduction

### Definition and classification of neurodegenerative diseases

Neurodegenerative diseases (NDDs) are a major public health issue characterized by progressive neuronal loss and functional impairment (Nguyen et al., 2021; Wang et al., 2025). On the basis of their core pathological characteristics, these diseases can be classified into proteinopathies and demyelinating diseases. Proteinopathies include Alzheimer’s disease (AD) and Parkinson’s disease (PD), which are characterized by abnormal aggregation of amyloid-β (Aβ) and tau proteins in AD and α-synuclein in PD (Xu et al., 2025a). Demyelinating diseases, such as multiple sclerosis (MS), are primarily characterized by the loss of myelin due to oligodendrocyte damage (Coutinho Costa et al., 2023). Globally, approximately 50 million people are affected by these diseases, and by 2040, the associated medical costs are estimated to exceed 2.8 trillion US dollars (Nandi et al., 2022; Wong et al., 2023).

### Existing treatments and neuromodulation techniques

The existing treatment methods for NDDs, such as AD, PD, and MS, mainly include pharmacological treatment, cognitive rehabilitation, and neuromodulation techniques. However, these approaches primarily target symptom relief, and effective disease-modifying therapies are currently lacking. Among pharmacological agents, acetylcholinesterase inhibitors and other drugs can improve cognitive function, but their long-term use may lead to drug resistance and adverse reactions (Emamzadeh and Surguchov, 2018; Hayes, 2019). Emerging methods such as gene therapy and immunotherapy are under investigation, but their safety and effectiveness still need further validation (Emamzadeh and Surguchov, 2018). Cognitive rehabilitation training can enhance cognitive function, but its effectiveness in these diseases has not been fully assessed (Gratwicke et al., 2015). More recently, targeted treatments for specific pathological features have become a research hotspot. For example, in AD, monoclonal antibodies targeting Aβ and tau protein have shown potential to slow cognitive decline in clinical trials, but their long-term and adverse effects still require further investigation (Xiao and Tan, 2024; Zhang et al., 2024e).

Neuromodulation, a biomedical engineering approach, modulates neuronal activity and neuroplasticity in the brain using electrical stimulation, magnetic fields, or ultrasound to treat and ameliorate diseases. Neuromodulation is critically important in the treatment of NDDs when medications become less effective. Currently, the core technologies in the field of neuromodulation include deep brain stimulation (DBS), transcranial magnetic stimulation (TMS), transcranial direct current stimulation (tDCS), and high-intensity focused ultrasound (HIFU), all of which have demonstrated conclusive efficacy in the treatment of NDDs. However, the clinical application of these technologies is still associated with some risks and limitations, which are summarized in **Additional Table 1**.

**Additional Table 1:** Summary of available neuromodulation techniques for the treatment of NDDs

Type of neuromodulation techniques	Site of action	Type of treatment/ effectiveness	Risk and limitation	Disease and efficacy	Reference
DBS	Basal ganglia (e.g., STN or GPi)	Significant improvement in motor symptoms such as tremor, muscle tone, and bradykinesia	Surgical implantation is needed, which carries a risk of infection and device malfunction. Additionally, periodic adjustments to the device parameters may be necessary, and the improvement in cognitive and axial symptoms tends to be relatively limited.	PD: Significant improvement in motor symptoms. AD: Limited studies, potential cognitive improvement. MS: Limited effectiveness for specific symptoms. FTD: Limited studies, effects not significant. MSA: Limited studies, effects not significant.	Emamzadeh and Surguchov, 2018; Hayes, 2019; Marín et al., 2022
TMS	Cortical surfaces (e.g., M1 area or DLPFC)	Potential improvement in motor and cognitive functions and attenuation of abnormal synchronized oscillations	Efficacy may be limited and ongoing treatment is needed.	PD: Potential improvement in motor and cognitive functions. AD: Limited studies, potential cognitive improvement. MS: Limited studies, effects not significant. FTD: Limited studies, effects not significant. MSA: Limited studies, effects not significant.	Khedr et al., 2019; Hill et al., 2020; Desarkar et al., 2024
tDCS	Cortical surfaces (e.g., M1 area or DLPFC)	Improve motor and cognitive functions and increase dopamine release	Efficacy may be limited and ongoing treatment is needed.	PD: Potential improvement in motor and cognitive functions. AD: Limited studies, potential cognitive improvement. MS: Limited studies, effects not significant. FTD: Limited studies, effects not significant. MSA: Limited studies, effects not significant.	Adenzato et al., 2019; Horiba et al., 2019; Prathum et al., 2025
HIFU	Specific brain area (e.g., Vim or STN)	Surgery for the treatment of tremor-predominant motor symptoms	Although this technique allows precise ablation of the target site without damaging other tissues, it is essentially an irreversible coagulative necrosis.	PD: Effective for tremor-predominant motor symptoms. AD: Limited studies, effects not significant. MS: Limited studies, effects not significant. FTD: Limited studies, effects not significant. MSA: Limited studies, effects not significant.	Obeso et al., 2017; Walters and Shah, 2019; Rollins et al., 2024
Spinal cord electrical stimulation	Medulla spinalis	Impact on pain and motor symptoms	Requiring implantable devices and there is limited effectiveness for specific symptoms.	PD: Limited effectiveness for specific symptoms. AD: Limited studies, effects not significant. MS: Limited effectiveness for specific symptoms. FTD: Limited studies, effects not significant. MSA: Limited studies, effects not significant.	Expert Consensus Group for Spinal Cord Stimulation for Chronic P, 2021; Prat-Ortega et al., 2025
Optogenetic techniques	Particular nerve cell	Show great potential in basic research for precise neuromodulation	Still in the early stages. The clinical application requires further research.	PD: Limited studies, potential for precise neuromodulation. AD: Limited studies, potential for precise neuromodulation. MS: Limited studies, potential for precise neuromodulation. FTD: Limited studies, potential for precise neuromodulation. MSA: Limited studies, potential for precise neuromodulation.	Imai et al., 2019; Zhang et al., 2024b
LIUS	Dopaminergic neurons, motor cortex, specific brain area (e.g., Vim or STN)	Deep penetration capability and neuroplasticity modulation capabilities	Unclear neurobiological mechanisms, technical limitations, and the impact of anatomical variations on treatment precision.	PD: Potential improvement in motor symptoms and cognitive function. AD: Limited studies, potential cognitive improvement. MS: Limited studies, potential for neuroplasticity modulation. FTD: Limited studies, effects not significant. MSA: Limited studies, effects not significant.	Yoo et al., 2011; Toccaceli et al., 2019; Wang et al., 2019b

The table compares LIUS with established neuromodulation techniques (DBS, TMS, tDCS, and HIFU) in terms of their site of action, treatment effectiveness, and associated risks/ limitations across various neurodegenerative diseases. Unlike DBS, which requires surgical implantation and carries infection risks, LIUS offers a noninvasive approach with DBP and neuroplasticity modulation. While TMS and tDCS are also noninvasive, their effects are limited primarily to cortical surfaces, whereas LIUS can target deeper brain structures. Although precise, HIFU causes irreversible tissue damage, which contrasts with the reversible and modulatory effects of LIUS. LIUS stands out for its balance of safety, efficacy, and feasibility, avoiding the invasiveness of DBS and the limitations of TMS/tDCS while offering a more controlled alternative to HIFU. However, further research is needed to optimize LIUS parameters and clarify their mechanisms. AD: Alzheimer's disease; DBS: deep brain stimulation; DLPFC: dorsolateral prefrontal cortex; FTD: frontotemporal dementia; GPi: globus pallidus internus; HIFU: high-intensity focused ultrasound; LIUS: low-intensity ultrasound stimulation; MS: multiple sclerosis; MSA: multiple system atrophy; NDDs: neurodegenerative diseases; PD: Parkinson's disease; STN: subthalamic nucleus; tDCS: transcranial direct current stimulation; TMS: transcranial magnetic stimulation.

### Mechanisms of low-intensity transcranial ultrasound

Transcranial ultrasound has been widely accepted and explored due to its noninvasive characteristics. Specifically, low-intensity transcranial ultrasound (LIUS) technology, with its ability to reversibly and bidirectionally regulate target neuron activity, has become a major highlight. Furthermore, LIUS also offers the advantages of high spatial resolution and high penetration. LIUS can improve neurodegeneration through multimodal mechanisms. At the molecular level, LIUS can inhibit M1 polarization of microglia, reducing neuroinflammation (Hu et al., 2024). At the cellular level, LIUS can activate the autophagy pathway to clear abnormal protein aggregation (Huang et al., 2021). At the systemic level, LIUS can enhance the functioning of the neurovascular unit (NVU), improving the microenvironment (Balzano et al., 2025). This broad-spectrum effect can potentially make LIUS a common treatment strategy for diseases such as AD, PD, and MS. For example, in AD models, LIUS has been shown to enhance the permeability of the blood–brain barrier (BBB) to clear Aβ (Rezai et al., 2024). In MS, it has been shown to accelerate myelin repair by promoting the differentiation of oligodendrocytes (Zhang et al., 2024a).

This review assesses the role of LIUS in the management of NDDs, detailing the mechanisms of action of low-intensity pulsed ultrasound stimulation (LIPUS: a specific type of ultrasound delivered at low intensity and output in a pulsed wave pattern) and low-intensity focused ultrasound stimulation (LIFUS: a type of focused ultrasound technology that uses a focused emission sound field). The effect of the sound beam converging along the sound axis during treatment is more pronounced than that of ordinary ultrasound. This discussion highlights the efficacy of these approaches in NDD models, as well as the benefits and challenges observed in animal studies and trials. Notably, the death of dopaminergic neurons is a central pathological hallmark of NDDs. We propose that LIUS may promote the regeneration of these neurons, presenting a novel therapeutic avenue. We hope that this review will provide a reference for the advancements and application of LIUS in neuromodulation.

### Purpose and innovations

This review explores the therapeutic potential and mechanisms of LIUS across several NDDs, such as AD, PD, MS, frontotemporal dementia (FTD), and multiple system atrophy (MSA). It compiles the latest research, addressing the capacity of LIUS to drive neuronal regeneration, enhance neural function, and regulate neuroinflammation in diverse disease models. The review explores the multifaceted mechanisms of LIUS, including neuroprotection and neuroregeneration, as well as the influence of its parameters, such as frequency, intensity, and duty cycle (DC), on treatment outcomes. Additionally, it evaluates the safety and efficacy of LIUS on the basis of animal studies and early-phase clinical trials, suggesting that LIUS can revolutionize the treatment of NDDs. By integrating animal study findings with clinical trial data, the review emphasizes the translational potential and safety of LIUS.

## Search Strategy

We conducted searches in the PubMed (September 2024 to February 2025), Google Scholar (from inception to February 2025), and Sci-Hub (from inception to February 2025) databases, utilizing the search terms listed in **Additional Table 2**. The search strategy outlined in **Additional Table 2** was used to ensure comprehensive article retrieval, followed by further screening through assessment of abstracts and titles to confirm that the retrieved articles were relevant to our target content. All cited articles were in English and, where possible, were the most recent. However, we also referenced a small number of older studies that traced the discovery and development of LIUS and NDDs.

**Additional Table 2:** Search strategy for the PubMed, Google Scholar, and Sci-Hub databases

Query	Search term
#1	Low-intensity pulsed ultrasound stimulation
#2	Low-intensity focused ultrasound stimulation
#3	Neuromodulation
#4	Neurodegenerative diseases
#5	Alzheimer's disease
#6	Parkinson's disease
#7	Multiple sclerosis
#8	Temporal lobe dementia
#9	Multiple system atrophy
#10	Neuronal regeneration
#11	#1 AND #3
#12	#2 AND #3
#13	#1 AND #3 AND #4
#14	#2 AND #3 AND #4
#15	#1 AND #3 AND #5
#16	#1 AND #3 AND #6
#17	#1 AND #3 AND #7
#18	#1 AND #3 AND #8
#19	#1 AND #3 AND #9
#20	#1 AND #3 AND #10
#21	#2 AND #3 AND #5
#22	#2 AND #3 AND #6
#23	#2 AND #3 AND #7
#24	#2 AND #3 AND #8
#25	#2 AND #3 AND #9
#26	#2 AND #3 AND #10

## Neurodegenerative Diseases and Cell Death Mechanisms

NDDs are closely associated with a variety of cell death mechanisms. Apoptosis triggers programmed neuronal death through pathways such as the mitochondrial or death receptor pathways (Yuan and Ofengeim, 2023). Necroptosis, which is mediated by proteins such as receptor-interacting serine/threonine-protein kinase 1 (RIPK1), receptor-interacting serine/threonine-protein kinase 3 (RIPK3), and mixed lineage kinase domain like pseudokinase (MLKL), leads to neuronal necrosis and inflammation (Dong et al., 2022). Ferroptosis is caused by iron metabolism disorders and lipid peroxidation, which destroy the cell membrane and cause cell death (Fujii and Imai, 2024). Cuproptosis is triggered by the accumulation of excessive copper within cells, causing mitochondrial dysfunction and leading to neuronal death (Fan et al., 2024). In addition, autophagy dysfunction is also related to neuronal death (Nixon and Rubinsztein, 2024).

### Alzheimer’s disease

In AD, a common neurodegenerative disorder marked by progressive cognitive and memory decline (Dziewa et al., 2024), the core pathology involving the buildup of tau protein tangles and Aβ plaques drives neuronal death and brain atrophy (Rahman and Lendel, 2021). Recent studies have highlighted various cell death mechanisms underlying AD, such as tau protein-linked apoptosis and ferroptosis, along with other novel forms of programmed cell death.

#### Tau protein and apoptosis

Tau protein, a microtubule-associated protein, is a key component of neurofibrillary tangles (NFTs) in AD (Moloney et al., 2021). The phosphorylation of tau protein by various kinases disrupts its normal function (Xia et al., 2021). The formation of toxic oligomers and aggregates promotes neurotoxicity, leading to the cognitive decline observed in patients with AD (Niewiadomska et al., 2021). This process is closely related to neuronal dysfunction and cell death. Additionally, studies have shown that the neuron-specific long non-coding RNA MEG3 is upregulated in AD patients, leading to neuronal necrosis (Plewka and Raczynska, 2022; Zheng and Wang, 2025). Downregulating MEG3 can inhibit neuronal death in AD mouse models.

#### Aggregation of amyloid-beta

The deposition of Aβ forms amyloid plaques outside neurons, which can trigger activation of microglia, leading to neuroinflammation (Merighi et al., 2022). Aggregation of Aβ can induce neuronal cell cycle re-entry through a tau-dependent mechanism, a process that leads to synaptic dysfunction and neuronal death (García-Osta et al., 2022; Liu et al., 2024). Cell cycle re-entry is considered an early key event in neuronal loss in AD (Qu et al., 2024).

#### Oxidative stress

The accumulation of reactive oxygen species (ROS) caused by metal ion imbalance and mitochondrial dysfunction leads to oxidative damage to lipids, proteins, and nucleic acids (Wang et al., 2020). Aβ plaques and tau aggregates can induce the production of ROS, causing neuronal death (Zhang et al., 2021a). The interaction between oxidative stress and other pathological mechanisms (such as tau protein phosphorylation and Aβ accumulation) highlights the complexity of AD pathology (Bhatt et al., 2021).

#### Ferroptosis and cuproptosis

Ferroptosis is a form of programmed cell death characterized by iron-dependent lipid peroxidation and excessive accumulation of ROS (Zhang et al., 2022). Recent studies have shown that ferroptosis is closely related to the onset, development, and prognosis of AD (Yan et al., 2021a; He et al., 2022). Imbalance of iron homeostasis, especially imbalance of the Xc^–^ system and glutathione peroxidase 4 (GPX4), plays a key role in this process (Shen et al., 2025). Iron accumulation leads to lipid peroxidation, exacerbating neuronal damage and promoting the pathological process of AD (Onukwufor et al., 2022).

Cuproptosis, a newly identified form of cell death triggered by copper metabolism dysfunction, has also been implicated in AD pathology (Wang et al., 2024). Excessive copper accumulation can interact directly with Aβ plaques and amyloid precursor proteins, worsening cognitive deficits (Squitti et al., 2021). While the precise mechanisms underlying cuproptosis in AD are still being studied, evidence suggests it may involve the buildup of fatty acylated proteins and disruption of iron-sulfur cluster proteins, both of which contribute to neuronal injury (Li et al., 2025).

#### Disulfidptosis

Disulfidptosis is an emerging form of programmed cell death caused by glucose starvation, leading to cysteine accumulation and disulfide stress (Ma et al., 2023). This process is linked to damage to the actin cytoskeleton, which is vital for normal neuronal function (Guo et al., 2024). While the exact role of disulfidptosis in AD is still under investigation, it is thought to represent a new pathway contributing to neuronal death.

### Parkinson’s disease

In PD, the degeneration of dopaminergic neurons, especially in the substantia nigra pars compacta, leads to reduced dopamine levels (Kalia and Lang, 2015). This process involves multiple programmed cell death pathways, including apoptosis, necrosis, and other regulated mechanisms, and is associated with both motor and non-motor symptoms.

#### Apoptosis

Neurodegeneration is influenced by apoptosis, a process managed by both intrinsic and extrinsic pathways. The intrinsic pathway, which is linked to mitochondria, is substantially affected by an imbalance in B-cell lymphoma (BCL)-2 family proteins (Czabotar and Garcia-Saez, 2023). This occurs when anti-apoptotic proteins such as BCL-2 and BCL-XL are downregulated and pro-apoptotic proteins such as Bcl-2 interacting mediator of cell death (BIM) and p53 upregulated modulator of apoptosis (PUMA) are upregulated (Bekker et al., 2021; Larrañaga-SanMiguel et al., 2025). These changes can result in mitochondrial membrane rupture, cytochrome c release, and activation of the caspase cascade, ultimately leading to neuronal death (Merighi and Lossi, 2022). Research on the SOD1G93A transgenic mouse model has shown that BIM deficiency can extend the mouse lifespan, while the combined absence of Bcl-2-like protein 4 (BAX) and Bcl-2 homologous antagonist/killer (BAK) can increase the neuronal count within the mouse brain (Baev et al., 2024).

The extrinsic apoptosis pathway acts through cell surface death receptors such as tumor necrosis factor receptor (TNFR) and Fas receptor, triggering a cascade that activates downstream caspase-8 and caspase-3 (Amin et al., 2022; Zhang et al., 2024d). The activation of tumor necrosis factor (TNF) and Fas signaling pathways in this process results in the release of inflammatory factors and cytokines. Chronic inflammation and immune responses can greatly enhance the extrinsic apoptosis pathway, exacerbating neuronal damage (Singh and Dikshit, 2007). Notably, serum levels of TNF-α in PD patients have been shown to correlate with disease severity, indicating the potential of TNF-α as a biomarker of disease progression (Qu et al., 2023). The interaction between the intrinsic and extrinsic apoptosis pathways can synergistically drive the death of dopaminergic neurons, accelerating the progression of PD.

#### Necroptosis

The mechanism of necroptosis in PD has not yet been fully elucidated. Unlike traditional apoptosis, necroptosis is a non-apoptotic form of cell death mediated by receptors. Necroptosis is usually activated by molecules such as RIP1 and RIP3 through signal transduction, which ultimately lead to cell membrane rupture and leakage of cell contents and thereby trigger local inflammatory responses (Zhang et al., 2024c). Dopaminergic neurons may initiate the necroptosis pathway as a result of oxidative stress, mitochondrial dysfunction, and excessive excitotoxicity, causing neuronal damage and exacerbating the progression of the disease (Gupta et al., 2023). In addition, the cell membrane rupture and inflammatory responses associated with necroptosis may exacerbate chronic inflammation in the nervous system, thereby promoting the development of the pathological process (Cai et al., 2024).

#### Ferroptosis

Ferroptosis is a recently characterized iron-dependent form of cell death that may play an important role in the neurodegenerative process of PD. Iron metabolism disorders are a key factor underlying ferroptosis, and excessive accumulation of iron not only promotes the generation of free radicals but also exacerbates oxidative stress and the accumulation of lipid-peroxidation products, thereby causing neuronal damage (Yao et al., 2024). In particular, the accumulation of iron in the substantia nigra is considered one of the main factors driving the pathological process (Ding et al., 2023).

The interplay between iron and lipid peroxidation products is a critical aspect of ferroptosis. Iron drives lipid peroxidation, producing substances such as linoleic acid peroxides. These substances then promote further iron buildup and activation, forming a loop that heightens cellular oxidative damage and impairs cell membrane integrity, eventually causing neuronal death (Sánchez-Alcázar et al., 2023; Cai et al., 2024). GPX4, a major antioxidant enzyme, combats this process by breaking down peroxidized lipids and stopping lipid peroxidation. However, reduced GPX4 activity leads to a buildup of lipid peroxides, intensifying ferroptosis and neuronal damage in PD (Dar et al., 2023).

#### Mitochondrial permeability transition-driven necrosis

Mitochondrial permeability transition (MPT)-driven necrosis plays an important role in PD by inducing cell necrosis through increased mitochondrial membrane permeability. The activation of MPT leads to an imbalance in ion concentrations inside and outside the mitochondria. These changes cause mitochondrial swelling and membrane rupture and release pro-apoptotic factors such as cytochrome c, a process that exacerbates neuronal damage (Guo et al., 2024). Mitochondrial dysfunction and excessive activation of MPT promote the progression of NDDs. Unlike the intrinsic apoptosis pathway, MPT-driven necrosis involves more structural damage to the mitochondrial membrane and cell membrane damage (Bernardi et al., 2023).

The role of cyclophilin D (CypD) in MPT-driven necrosis is mainly realized by regulating the opening of the mitochondrial permeability transition pore (mPTP) (Gutiérrez-Aguilar and Baines, 2015). Genetic knockout of CypD can reduce the opening of the mPTP, reducing the release of cell death factors such as cytochrome c and thereby protecting neurons from damage (Winquist and Gribkoff, 2020). In addition, the activity of CypD can be regulated by a variety of factors, such as intracellular calcium ion concentration, ROS levels, and mitochondrial membrane potential, and its role in MPT-driven necrosis is based on a complex regulatory mechanism (Carraro and Bernardi, 2016).

#### Roles of kinases, poly [ADP-ribose] polymerase 1, and tripartite motif-containing protein 31 in Parkinson’s disease

Excessive activation of kinases, poly [ADP-ribose] polymerase 1 (PARP1), and tripartite motif-containing protein 31 (TRIM31) influences the survival and function of neurons. A study on kinases indicated that RIPK1, which plays a pivotal role in the TNFR1 signaling pathway, can both promote the activation of nuclear factor-κappa B (NF-κB) and mitogen-activated protein kinase (MAPK) genes to sustain cell survival and induce apoptosis upon activation (Kim et al., 2023a). RIPK3, as the upstream kinase of MLKL, triggers necrosis after activation, and its inhibition can reduce microglial activation and protect dopaminergic neurons (Yañez et al., 2021). Ataxia-telangiectasia mutated (ATM) activates p53 in the DNA damage response, promoting the apoptotic pathway and increasing cell sensitivity to oxidative stress (Gonzalez‐Hunt and Sanders, 2020). CDK5 inactivates the catalase Prx2 in PD models, blocking DNA repair and contributing to pathology (Qu et al., 2007). The overexpression of leucine-rich repeat kinase 2 (LRRK2) induces apoptosis, activates multiple kinases, and inhibits neuronal survival (Abshana et al., 2023). Apoptosis signal-regulating kinase 1 (ASK1) activates several apoptotic pathways, and its expression increases in the brains of patients with PD (Mansour et al., 2023).

In addition, excessive activation of PARP1 not only induces apoptosis but also leads to energy-metabolism disorders, affects mitochondrial function, exacerbates oxidative stress damage, and regulates gene expression, affecting the plasticity and synaptic function of neurons and thereby affecting the cognitive and motor functions of patients with PD (Mao and Zhang, 2021). TRIM31, through its E3 ubiquitin ligase activity, interacts with voltage-dependent anion-selective channel 1 (VDAC1) and catalyzes K48-linked polyubiquitination, promoting the proteasomal degradation of VDAC1 and thereby playing an important role in maintaining the homeostasis of DA neurons. Absence of TRIM31 leads to an increase in VDAC1 protein levels, exacerbating neuronal death in PD models (Meng et al., 2024).

**Figure 1:**
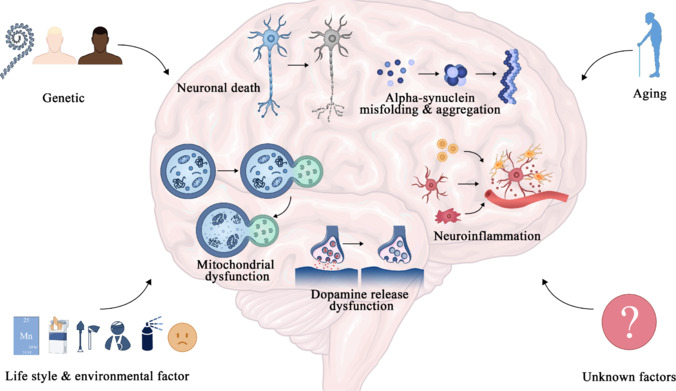
Mechanisms of Parkinson’s disease.

The mechanisms of PD are illustrated in **Figure 1**. PD is characterized by the aggregation of α-synuclein into Lewy bodies and neurites, which is pathognomonic for the condition (Reich and Savitt, 2019). The global prevalence of PD is rising, particularly among older adults, with the disease affecting over 1% of individuals over the age of 60 years (Reeve et al., 2014). The incidence of PD also shows racial variations, with White populations showing higher rates than African Americans (Dahodwala et al., 2009). Various lifestyle, environmental, and genetic factors contribute to susceptibility to PD; risk factors include constipation, head trauma, anxiety, depression, and pesticide exposure, while physical activity and caffeine intake may help mitigate this risk (Bellou et al., 2016). The significant genetic risk factors for PD include mutations in the *SNCA* and *LRRK2* genes, as well as the *GBA* gene, which is associated with Gaucher disease (Bloem et al., 2021).

### Multiple sclerosis

MS is a chronic inflammatory disease characterized by demyelination in the central nervous system (CNS) (Papiri et al., 2023). Its pathological features include inflammatory cell infiltration, oligodendrocyte reduction, and neuronal damage (Pukoli and Vécsei, 2023). Recent research has revealed the roles of various cell death mechanisms in MS, including ferroptosis, neuroinflammation, and necrosis.

#### Ferroptosis and T cell activation

Studies have shown that ferroptosis is closely related to the inflammatory infiltration and demyelination processes in MS (White, 2022; Jhelum et al., 2023). MS patients show various signs of ferroptosis in active and chronic lesions, as well as in the cerebrospinal fluid, including unstable iron levels and increased levels of peroxidized phospholipids and lipid degradation products (Wei and Jian, 2025). Ferroptosis can drive T cell activation via T cell receptor signaling, contributing to neurodegeneration (Zeng et al., 2024). In an experimental autoimmune encephalitis mouse model, using ferroptosis inhibitors or reducing acyl-CoA synthetase long-chain family member 4 (ACSL4) expression significantly delayed relapse and improved disease progression (Luoqian et al., 2022).

#### Neuroinflammation

In neurodegenerative disorders, Ninjurin 1 (NINJ1), a protein implicated in cell death and inflammation, can heighten BBB permeability, facilitating the entry and activation of inflammatory cells within the CNS (Xu et al., 2024). NINJ1 exacerbates plasma membrane rupture during cell death, leading to the leakage of inflammatory mediators that can damage nearby tissue (Kayagaki et al., 2021). Additionally, the activation of the NF-κB signaling pathway plays a significant role in the inflammation associated with MS, linking inflammation to neuronal damage (Yan et al., 2021b).

#### Necrosis

Signaling pathways involving RIPK1 and RIPK3 have been shown to regulate the death of oligodendrocytes *in vivo* (Zelic et al., 2021). Upregulation of RIPK1 and caspase-independent cell death have been observed in cuprizone-induced mouse models of oligodendrocyte demyelination (Zirngibl et al., 2022). RIPK1 inhibitors can effectively inhibit the death of oligodendrocytes and inhibit TNF-α-induced oligodendrocyte necrosis *in vitro* (Ma et al., 2022).

### Frontotemporal dementia and cell death mechanisms

FTD is the term used to refer to a group of NDDs characterized by behavioral and language disorders (Geraudie et al., 2021). The pathological features of FTD include neuronal death, neuroglial cell proliferation, and protein aggregation (Hartnell et al., 2021). Recent studies have revealed the roles of various cell death mechanisms in FTD, including ferroptosis, neuroinflammation, necrosis, and RNA-splicing abnormalities.

#### Ferroptosis

In FTD, ferroptosis, a form of cell death triggered by the oxidation of polyunsaturated fatty acids catalyzed by iron, is characterized by lipid peroxidation and the accumulation of ROS, which ultimately lead to cell membrane destruction and cell death (Ferretti and Zanella, 2024). Iron metabolism disorders and lipid peroxidation are widespread in the brain of patients with FTD, and the accumulation of iron can trigger the Fenton reaction, producing hydroxyl radicals that directly react with cell membranes and lipids, leading to oxidative stress and cell death (Gorini and Tonacci, 2024). Furthermore, an imbalance in the amino acid antioxidant system (such as system xc^–^) can exacerbate lipid peroxidation and promote ferroptosis (Rochette et al., 2022). Key enzymes involved in lipid peroxidation, including ACSL4 and lysophosphatidylcholine acyltransferase 3 (LPCAT3), are upregulated in FTD, which promotes the formation of lipid peroxides and the progression of ferroptosis (Long et al., 2023).

#### Oxidative stress

Elevated levels of ROS and impaired oxidative phosphorylation observed in the brains of FTD patients lead to oxidative damage to lipids, proteins, and nucleic acids, impairing cell function and viability (Anoar et al., 2021). The transcription factor nuclear factor erythroid 2-related factor 2 (NRF2), which regulates cellular redox homeostasis, plays a crucial role in protecting brain cells against oxidative stress and ferroptosis (Brackhan et al., 2023). Additionally, oxidative stress promotes the aggregation of proteins such as microtubule-associated protein tau (MAPT/TAU) and transactive response DNA binding protein 43 kDa (TARDBP/TDP43), which are commonly found in FTD-associated inclusions, further contributing to neurotoxicity and neuronal death (Ferretti and Zanella, 2024).

#### Neuroinflammation

In the brains of patients with FTD, the upregulation of inflammation-related genes such as *C3*, *SPARC*, and *SPP1* (osteopontin [OPN]) can trigger inflammatory responses in microglia, leading to neuronal death (Al-Dalahmah et al., 2024). Additionally, OPN, a proinflammatory factor, can cause inflammatory responses in microglia, leading to increased expression of related factors such as TNF, microphthalmia-associated transcription factor (MITF), glycoprotein NMB (GPNMB), and C–X–C chemokine receptor type 4 (CXCR4) (Bonham et al., 2018; Pang and Hu, 2020; Clarke et al., 2024).

#### Necroptosis

Necroptosis, a regulated form of necrosis that occurs when apoptosis is inhibited, is characterized by cell swelling, membrane rupture, and the release of damage-associated molecular patterns (DAMPs) such as mitochondrial DNA (mtDNA), high mobility group box 1 (HMGB1), and interleukin (IL)-1α, IL-33, and ATP, and is marked by the activation of key proteins, including RIPK1, RIPK3, and MLKL (Frank and Vince, 2018). When caspase-8 activity is inhibited, RIPK1 recruits and activates RIPK3, which in turn phosphorylates MLKL, causing the phosphorylated MLKL to translocate to the plasma membrane and form pores that lead to membrane permeability and cell destruction (Mitroshina et al., 2023). The release of DAMPs during necroptosis triggers inflammation, contributing to the neurodegeneration observed in FTD (Clark and Vissel, 2025).

#### RNA-splicing abnormalities

Al-Dalahmah et al. (2024) found that the functioning of nuclear speckles is disrupted in the brains of patients with FTD, resulting in widespread changes in RNA alternative splicing, which particularly influence exon skipping and intron retention events. The loss of core components of nuclear speckles, such as SRRM2 and SON, leads to significant neuronal death; however, overexpression of SRRM2 can partially reverse the cell death caused by nuclear speckle dysfunction (Li and Sun, 2025).

### Multiple system atrophy

MSA is a rare NDD characterized by autonomic dysfunction, Parkinsonism, cerebellar ataxia, and pyramidal tract signs (Stankovic et al., 2022). The pathological mechanisms underlying MSA are not fully understood, but studies have shown that it is a primary oligodendroglial disease involving various cell death mechanisms, including abnormal aggregation of α-synuclein, neuroglial cell lesions, mitochondrial dysfunction, and immune-inflammatory responses (Stefanova and Wenning, 2023; Wang et al., 2023a, b).

#### Abnormal aggregation of α-synuclein and neuroglial cell pathology

The misfolding and aggregation of α-synuclein into Lewy body-like inclusions damage neurons and disrupt cellular functions and signaling (Koga et al., 2021). These inclusions also interfere with neuronal support, activate resting microglia, and induce progressive oligodendrocyte dysfunction (Ndayisaba et al., 2025). Pathological changes in oligodendrocytes, including the formation of eosinophilic inclusions, further impair normal neuronal function and trigger neurodegeneration (Sian-Hulsmann and Riederer, 2023). p25α, a key stabilizer of myelin integrity, redistributes to the oligodendrocyte soma before the appearance of α-synuclein deposits. This is followed by oligodendrocyte swelling and abnormal α-synuclein uptake and overexpression (Ferreira et al., 2021; Stefanova and Wenning, 2023). Collectively, these changes undermine neuronal support and exacerbate neuronal death.

#### Mitochondrial dysfunction and immune-inflammatory response

Mitochondrial dysfunction results in insufficient energy production, disrupting intracellular homeostasis and leading to cell damage and death, participating in the pathological process of MSA (Krismer and Wenning, 2017). Mitochondrial dysfunction may be related to the aggregation of α-synuclein, further exacerbating oxidative stress and energy metabolic disorders in cells (Cheng et al., 2021). Immune dysfunction, release of inflammatory factors, activation of microglia, and the resulting inflammatory response cause damage to nerve cells (Ma et al., 2024). Neuroinflammatory responses, the absence of oligodendrocyte support for neuronal nutrition, and neuronal functional abnormalities caused by α-synuclein act synergistically to promote neuronal death and subsequent reactive astrocytosis (Motyl et al., 2024).

## Transcranial Ultrasound in Neural Repair and Regeneration

By harnessing the mechanical effects of sound waves, transcranial ultrasound can modulate neuronal activity to facilitate neuronal growth, repair, and functional recovery. These effects influence several key areas: enhancing the differentiation of neural progenitor cells and stem cells, facilitating NVU repair, adjusting CNS plasticity, and preserving neurotransmitter balance.

### Neural progenitor and stem cell differentiation

Transcranial ultrasound can utilize the mechanical properties of sound waves to activate mechanosensitive ion channels on cell membranes, thereby inducing depolarization or hyperpolarization. This process is closely associated with the activation of voltage-gated sodium (Na^+^) and calcium (Ca^2+^) channels, which are crucial for promoting neurite growth and strengthening neural connections (Burks et al., 2019; Pellow et al., 2024). Through these mechanisms, transcranial ultrasound can effectively stimulate the proliferation and differentiation of neural progenitor and stem cells, facilitating neural repair and regeneration. It can also regulate BBB permeability, activate endogenous neural stem cells, upregulate neural stem cell markers such as Sox2 and nestin, and enhance the expression of neurotrophic factors such as brain-derived neurotrophic factor (BDNF), all of which support neural stem cell differentiation (He et al., 2021; Seo et al., 2023). Furthermore, the focal adhesion kinase (FAK)-extracellular signal-regulated kinase 1/2 (ERK1/2) signaling pathway has been confirmed to play a role in this neurogenesis process (Wang et al., 2022b).

### Regulation of neurovascular units

Transcranial ultrasound promotes angiogenesis and enhances cerebral blood flow, creating an environment conducive to neural regeneration. Beyond its effects on neurons and neural stem cells, it exerts a substantial influence on NVUs. By activating the vascular endothelial growth factor signaling pathway, it stimulates vascular neogenesis and boosts cerebral blood flow, ensuring the delivery of more oxygen and nutrients to support neural regeneration (Su et al., 2017; Yi et al., 2024). Additionally, transcranial ultrasound reduces inflammation and oxidative stress, protecting neurons and vascular endothelial cells. This protective effect facilitates the repair and regeneration of NVUs. For example, one study demonstrated that transcranial ultrasound treatment decreased inflammatory cell infiltration and oxidative stress in a traumatic brain injury rat model, promoting the repair and regeneration of NVUs (Huang et al., 2022).

### Central nervous system regulation and peripheral nerve injury repair

Transcranial ultrasound has broad application prospects in the regulation of the CNS and the repair of peripheral nerve injuries. In terms of CNS regulation, transcranial ultrasound can modulate emotions and pain. For instance, transcranial ultrasound stimulation to the posterior frontal lobe in patients with chronic pain yielded improvements in both emotional and pain scores (Pellow et al., 2024). In brain injury animal models, LIPUS has been shown to inhibit the reduction of BDNF expression caused by bilateral carotid artery occlusion and reduce neuronal damage and demyelination of nerve fibers (Huang et al., 2017). LIPUS can also reduce the memory impairment caused by chronic neuroinflammation by inhibiting the Toll-like receptor 4/NF-κB pathway and enhancing BDNF expression (Chen et al., 2019b). In terms of peripheral nerve injury repair, postoperative application of LIPUS can promote nerve regeneration and improve neuromuscular innervation. Schwann cells, the supporting glial cells of peripheral nerves, can convert to a “repair” phenotype to coordinate the response to nerve injury. Two of the existing studies have focused on promoting and expanding the repair function of Schwann cells (Yi et al., 2023; Zhong et al., 2023).

### Effects of transcranial ultrasound on neurotransmitters and neural networks

Transcranial ultrasound modulates neuronal activity and neural network dynamics, altering the levels of neurotransmitters such as dopamine and serotonin and thereby influencing behavior and cognitive functions (Wang et al., 2019a). It also enhances brain plasticity, supporting recovery and functional rehabilitation following neural injury (Gupta, 2023). By optimizing the balance of neuronal excitation and inhibition and enhancing cerebral cortex neural network function, this therapeutic modality can facilitate neural repair and accelerate functional recovery. Moreover, transcranial ultrasound can regulate neurotransmitters such as glutamate and GABA, enhancing neuronal conduction and synaptic transmission, thereby promoting the remodeling of neural networks. (Yaakub et al., 2023).

### Combined application of transcranial ultrasound with other neuromodulation techniques

Transcranial ultrasound can be combined with other neuromodulation techniques and therapeutic approaches to enhance neural repair and regeneration. For instance, when paired with TMS, this technique can improve functional recovery after neural injury by upregulating neuroplasticity in the brain (Chen et al., 2024a). This combination therapy, which leverages two distinct mechanisms, works synergistically to help the brain reconfigure neural networks and promote recovery in damaged regions. Additionally, the combination of transcranial ultrasound with mesenchymal stem cell therapy has been shown to increase cell survival rates and drive neural repair and regeneration (Wu et al., 2025). Similarly, studies suggest that combining transcranial ultrasound with nerve growth factor or drug therapies can enhance the neural repair process and support the growth and survival of neurons (Xhima and Aubert, 2021; Aryal et al., 2022).

## Mechanisms Underlying the Effects of Low-Intensity Transcranial Ultrasound in Neurodegenerative Diseases

NDDs, such as AD and PD, are characterized by neuronal damage, neuroinflammation, oxidative stress, and neuronal death. LIUS, as an emerging therapeutic approach, has shown substantial potential for treating NDDs. LIUS provides new avenues for the treatment of AD by reducing Aβ deposition, enhancing neural plasticity, regulating neuroinflammation, temporarily opening the BBB, and enhancing neurotrophic factors and synaptic plasticity.

### Mechanisms underlying the effects of low-intensity transcranial ultrasound in Alzheimer’s disease

#### Promotion of neurite outgrowth and upregulation of neurotrophic factors

Neuronal dysfunction, which is one of the early pathological features of AD, involves a variety of mechanisms, including neurotransmitter synthesis, cellular and vesicular uptake, action potential conduction, and the role of local neuromodulatory receptors (Chowdari Gurram et al., 2024). LIUS promotes neurite outgrowth and enhances the expression of neurotrophic factors, facilitating neuroprotection and neural repair/regeneration. A previous study demonstrated that ultrasound influences cell proliferation and differentiation by inducing the release of intracellular nitric oxide, which, in turn, promotes neurite growth (Yang et al., 2023). LIUS activates intracellular pathways (such as ERK1/2-cyclic adenosine monophosphate response element binding protein [CREB]-thioredoxin 1 [Trx-1]) through mechanotransduction, enhancing neurite outgrowth induced by nerve growth factors (such as BDNF and nerve growth factor [NGF]) (Ye et al., 2023). BDNF, as a crucial neurotrophic factor, participates in neuronal survival, differentiation, synaptic plasticity, and synapse formation, alleviating neurodegenerative processes and promoting the repair and functional recovery of damaged neurons (Kumari et al., 2023).

LIUS can modulate neuronal excitability by activating mechanosensitive ion channels, thereby triggering a series of intracellular reactions. These include transient changes in sodium and calcium ion levels, vesicle exocytosis, and synaptic transmission. In turn, these processes elevate the levels of neurotrophic factors and foster neural repair and regeneration (Oyovwi et al., 2025). Moreover, LIUS can enhance neural plasticity and improve brain network connectivity, boosting the brain’s ability to adapt and recover (Yang et al., 2021).

#### Inhibiting inflammatory responses and oxidative stress

Neuroinflammation, which is a hallmark of AD, is characterized by the production of proinflammatory cytokines from activated microglia and astrocytes (Tramontin et al., 2021). LIUS can modulate microglial activation to mitigate neuroinflammation, reducing the expression of proinflammatory factors such as TNF-α and IL-1β, which, in turn, reduces neurotoxicity and enhances neuroprotection (Li et al., 2023). Furthermore, LIUS can curb neuroinflammation through downstream signaling pathways such as phosphoinositide 3-kinase (PI3K)-protein kinase B (Akt) and ERK1/2 (Yu and Luo, 2024).

LIUS can reduce the accumulation of ROS and protect the mitochondrial membrane potential, thereby alleviating oxidative stress (Karthika et al., 2024). Oxidative stress is one of the main pathological processes in NDDs. LIUS can help slow down neurodegeneration by regulating the expression of antioxidant proteins and reducing intracellular oxidative stress levels (Wu et al., 2023).

#### Temporarily opening the blood–brain barrier

The BBB is a barrier that protects the brain from the entry of external substances, including drugs (López-Aguirre et al., 2024). The combination of LIUS and intravenous microbubbles (MB) can effectively, safely, and reversibly open the BBB, allowing noninvasive targeted drug delivery (Rafati et al., 2024). This mechanism has been validated in AD clinical trials. For example, a study by Feng et al. (2024) showed that LIUS can temporarily reduce BBB function and promote the entry of therapeutic agents into the brain.

### Mechanisms underlying the effects of low-intensity transcranial ultrasound in Parkinson’s disease

In PD therapeutic research, LIPUS and LIFUS have shown notable potential, mediating neurite outgrowth, enhancing neurotrophic factors, and suppressing neuroinflammatory and oxidative stress responses, offering novel pathways for neural repair and protection.

#### Promotion of neurite outgrowth and upregulation of neurotrophic factors

LIUS promotes neurite outgrowth and neurotrophic factor expression, mediating neuroprotection and neural repair/regeneration in NDDs. Fan et al. (2016) showed that ultrasound influences cell proliferation and differentiation by inducing the release of intracellular nitric oxide, thereby promoting neurite growth. LIPUS activates intracellular pathways such as ERK1/2-CREB-Trx-1 via mechanotransduction, enhancing NGF-induced neurite outgrowth (Zhao et al., 2016).

LIPUS and LIFUS enhance the protective and reparative functions of neurons by upregulating the expression of neurotrophic factors (such as BDNF and NGF). BDNF, as a key neurotrophic factor, is involved in neuronal survival, differentiation, synaptic plasticity, and synaptogenesis. Neuronal function is often impaired due to a lack of BDNF, and LIUS helps to mitigate neurodegenerative processes and promote the repair and functional recovery of damaged neurons by increasing BDNF expression (Zhong et al., 2023). Lin et al. (2015) found that LIPUS could increase the expression of BDNF in the hippocampus of AD model rats, effectively protecting neurons from damage.

Furthermore, LIPUS and LIFUS have been shown to modulate neuronal excitability by activating mechanosensitive ion channels, thereby modulating neuronal excitability. The activation of these channels can trigger a series of intracellular reactions, including transient changes in sodium and calcium ions, vesicle exocytosis, and synaptic transmission, all of which can enhance neurotrophic factor levels and promote neural repair and regeneration (Guerra and Bologna, 2022; Truong et al., 2022).

#### Inhibition of inflammatory responses and oxidative stress

LIUS can reduce the accumulation of ROS and protect mitochondrial membrane potential, thereby alleviating oxidative stress. Oxidative stress is a key pathological process in NDDs, and LIUS can reduce the oxidative stress levels within cells by regulating the expression of antioxidant proteins, helping to slow down neurodegeneration. For example, Sung et al. (2022) demonstrated that LIUS can reduce the production of proinflammatory factors such as TNF-α and IL-1β, which may help modulate overactivation of microglia. Through this process, LIUS may reduce neurotoxicity by alleviating oxidative stress and enhancing neuroprotective effects. Additionally, Song et al. (2022) reported that LIUS alleviated the dopaminergic neuronal damage induced by MPTP in PD models, promoting neural repair. In another study, Karmacharya et al. (2016) showed that LIUS could also suppress the ROS generation induced by 1-methyl-4-phenylpyridinium (MPP^+^), thereby inhibiting the aggregation of α-synuclein in PC12 cells.

In PD models, LIPUS has been shown to alleviate neuroinflammation and enhance neuroprotection by downregulating the expression of lipocalin-2 (Kim et al., 2023b). In contrast, Zhong et al. (2023) reported that LIFUS finely regulated neuroinflammatory responses by regulating the activation state of microglia. LIFUS was shown to modulate the neuroinflammatory response through inhibition or activation of microglia, which influenced neuroprotection.

LIPUS and LIFUS activate multiple signaling pathways that are sensitive to mechanical stress and pressure changes, converting ultrasound-induced physical stimuli into intracellular signals that dampen neuroinflammation. Studies have shown that LIUS may modulate downstream intracellular signal transduction and reduce the occurrence of neuroinflammation through pathways such as the PI3K-Akt pathway, ERK1/2 pathway, K2P channels, and mechanogated ion channels (Vallée et al., 2021; Ye et al., 2023).

### Potential mechanisms underlying the effects of low-intensity transcranial ultrasound in other neurodegenerative diseases

LIFUS and LIPUS have shown some initial progress in the field of neuromodulation, primarily focusing on NDDs, nerve repair, and cognitive impairments. However, research on diseases such as MS, FTD, and MSA is still in a very preliminary stage. The specific treatment mechanisms for these diseases remain unclear, and relevant research is relatively scarce. The existing studies have rarely covered the specific applications in these diseases, with most studies focusing on healthy individuals or other more common NDDs (Zhao et al., 2016; Kumari et al., 2023). Therefore, future studies should urgently aim to explore the mechanisms of action of LIUS in these specific diseases, characterize the potential therapeutic effects, and provide new treatment options for patients.

MS is a chronic inflammatory demyelinating disease of the CNS (Huang et al., 2023). LIUS, by promoting local blood flow, may help slow the demyelination process. Additionally, through neuromodulation, ultrasound may improve motor and cognitive functions in patients with MS. In particular, LIUS holds substantial potential as an adjunctive therapeutic method for nerve repair and regeneration, especially in improving motor and sensory functions in MS patients.

FTD is usually accompanied by neuronal death and neurofunctional degeneration (McCauley and Baloh, 2018). LIUS may delay the neurodegenerative process by improving local blood flow and promoting the release of neurotrophic factors, and may also improve cognitive and memory functions. Specifically, by stimulating neuroplasticity in the hippocampus and temporal lobe regions, LIUS can provide therapeutic effects and offer new treatment options for FTD patients.

MSA is a disease involving neuronal degeneration and multi-system functional loss (Donze et al., 2024). LIUS may alleviate or temporarily reduce some functional impairments in patients with MSA through its role in promoting neuroplasticity and repair. Although research on this topic is limited, the application of LIUS in NDDs still has certain exploratory value.

## Physical Parameters Influencing the Biological Effects

In LIUS, electrical energy is transformed into acoustic waves through mechanical deformation of piezoelectric crystals; these waves are then propagated through a medium and converted into biochemical signals, thereby affecting the physiological functions of cells and tissues (Khanna et al., 2008; del Rosario-Gilabert et al., 2024). The therapeutic effects of LIUS are influenced by several physical parameters, and a detailed analysis of these key physical parameters and their influence on the treatment of NDDs is provided below.

The fundamental frequency (FF), which typically ranges from 0.22 to 3.8 MHz in the treatment of NDDs, is a crucial parameter that determines the penetration depth and range of action of ultrasound waves. The specific frequency selected depends on the therapeutic target and the precise design of the equipment (Jiang et al., 2019). Low-frequency ultrasound (e.g., 0.5 MHz) can penetrate deeply to reach areas such as the subthalamic nucleus, globus pallidus interna, and pedunculopontine nucleus. In contrast, higher frequencies (e.g., 3 MHz and above) are primarily used for treatments targeting superficial structures, such as the ventrointermediate nucleus and parafascicular thalamus.

Spatial-peak temporal average (ISPTA) or spatial-peak pulse average (ISPPA) are fundamental parameters of LIUS, and are typically maintained between 0.02 and 1 W/cm² to ensure non-thermal effects during the treatment of NDDs (Feril et al., 2008). Depending on the therapeutic requirements, this intensity can be moderately increased to 5 W/cm^2^ within a safe range to enhance the penetration ability and therapeutic effects of the ultrasound waves (Bancel et al., 2024). Lower-intensity ultrasound is suitable for treating superficial tissues, while higher-intensity ultrasound is appropriate for deep tissue treatments, such as improving blood flow and promoting tissue repair. In studies by Uddin et al. (2021), although the thermal effect of LIUS was weak, its slight heating effect contributed positively to promoting blood flow and tissue repair.

The DC represents the ratio of the ultrasound emission time to the total cycle period. A higher DC increases the continuous action of ultrasound waves. By properly adjusting the range of DCs, appropriate cavitation effects can be produced to enhance the penetration of ultrasound treatment, help open the BBB, destroy pathological cells, and facilitate neural modulation (Meairs and Alonso, 2007; Mason, 2011; Polat et al., 2011). During drug treatment, microstreaming effects complement cavitation effects, jointly promoting local stress and cell membrane loosening, and facilitating nutrient exchange and drug delivery (Ennasr et al., 2024).

The mechanical index (MI) is an important parameter for assessing therapeutic and cavitation effects. At high MI values, the cavitation effect of ultrasound waves is enhanced, which facilitates the relaxation of cell membranes and increases their permeability, promoting drug delivery and enhancing cell repair capabilities (Kollmann et al., 2013). In the treatment of NDDs, cavitation effects are particularly important, since they can enhance the precision of treatment through minimally invasive means (Sen et al., 2015).

The pulse repetition frequency (PRF) directly influences the mechanical effects of ultrasound waves. A higher PRF (e.g., 500–1000 Hz) can boost cavitation effects and cell vibrations, increasing cell membrane permeability and promoting drug delivery, cell repair, and tissue regeneration (Fomenko et al., 2020). Conversely, a lower PRF is better suited for gentle treatments to prevent excessive cell activation.

The mark duration (MD) and space duration (SD) are key parameters that govern the duration and frequency of ultrasound treatment. MD refers to the duration of each pulse, while SD is the interval between pulses. Taken together, these factors influence the signal strength and periodic changes in ultrasound waves. Research indicates that varying MD and SD combinations can influence treatment outcomes. A shorter MD and longer SD reduce the continuous ultrasound action, preventing excessive tissue stimulation (Saber and Saber, 2017; Zhang et al., 2023; He et al., 2024). Conversely, a longer MD and shorter SD enhance treatment persistence and effectiveness, particularly in addressing deep or complex pathological conditions.

**Figure 2:**
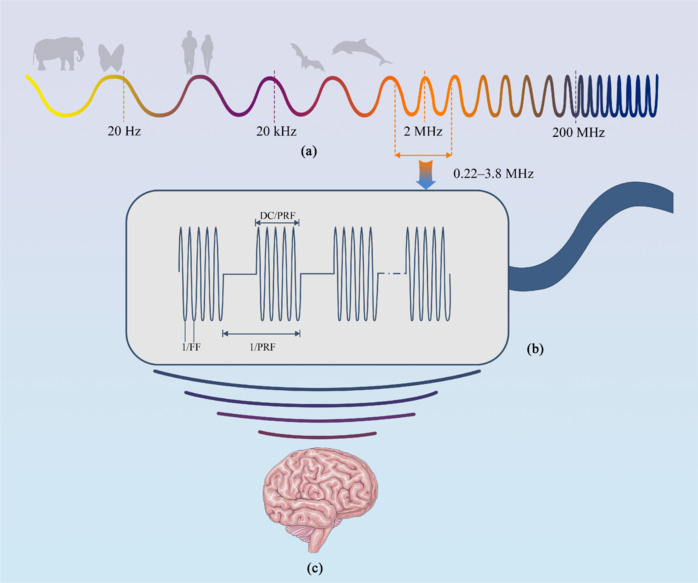
Low-intensity ultrasound stimulation (LIUS). Panel (a) displays the ultrasound frequency range, with LIUS typically using frequencies between 0.44 and 3.8 MHz. Panel (b) presents a schematic representation of the LIUS waveform. Panel (c) shows a schematic of the LIUS device in action on the human brain. DC: Duty cycle; PRF: pulse repetition frequecy.

The focal depth (FD) is a key parameter that controls the depth of focus of ultrasound energy. By accurately setting the FD, the energy of ultrasound waves can precisely target the treatment area, avoiding damage to surrounding healthy tissues (Padilla and ter Haar, 2022). In deep tissue treatments, setting the FD is particularly important to ensure the efficiency and accuracy of the therapy. Low-intensity ultrasound stimulation is illustrated in **Figure 2**.

## Animal Experiments and Clinical Applications

### Animal experiments

Animal experiments for LIPUS date back to 1972, when Taylor and Pond (1972) irradiated the spinal cord of adult rats with ultrasound (ISPPA = 25 or 50 W/cm^2^, FF = 0.5–6.0 MHz). To avoid thermal effects, the energy delivery was pulsed. In most experiments, the ratio of MD to SD (M:S) is usually 10:100 ms, which indicates that each pulse lasts for 10 ms and is followed by a 100-ms interval. This treatment was found to result in paraplegia or hemorrhage of the spinal cord. The appearance of hemorrhage was found to be more consistent and was used to compare the effects of different ultrasound parameters. The damage ability was the greatest at the lowest frequency used (0.5 MHz) and decreased as the frequency increased to 5 MHz, at which point neither paraplegia nor hemorrhage occurred.

The biological applications of LIFUS date back to 1942, when Lynn et al. (1942) experimentally used high-frequency ultrasound (FF = 835 kHz) to generate focused ultrasound by driving a quartz crystal. The equipment used in their experiments included a radio transmitter, an ultrasonic generator, and a transparent phenolic container that facilitated focusing of ultrasound waves onto biological materials. Studies conducted during this period examined the effects of ultrasound on various substrates, including oil films, paraffin, animal tissues, and live animals. The findings demonstrated the ability of ultrasound to induce targeted thermal and mechanical effects, thereby allowing for controlled tissue damage. These results indicate that focused ultrasound can produce localized damage in fresh tissues and living organisms, paving the way for potential applications in the biomedical field.

#### Applications of low-intensity transcranial ultrasound in animal models

LIPUS has been shown to increase the expression of neuroprotective factors, enhance cognitive function, and mitigate neuroinflammation and oxidative stress. In models of AD, LIPUS has been shown to elevate the levels of BDNF and glial cell line-derived neurotrophic factor, thereby protecting neurons (Zhang et al., 2023). It has also been shown to improve cerebral blood flow and reduce Aβ deposition by modulating the expression of endothelial nitric oxide synthase, which helps alleviate cognitive deficits (Shindo et al., 2024). In models of vascular dementia (VD), LIPUS promotes the proliferation and maturation of oligodendrocyte precursor cells, reduces white matter damage, improves cognitive function, and simultaneously increases vascular endothelial cell proliferation and angiogenesis. These effects can enhance cerebral blood flow and alleviate cognitive impairments (Eguchi et al., 2018). Identifying innovative neuroprotective approaches is essential for slowing the progression of NDDs. In a study using 6-hydroxydopamine-induced PD mouse models, LIPUS stimulation was applied to evaluate its effects on neuroinflammation, neurotrophic factor expression, BBB integrity, and dopamine transporter expression (Song et al., 2022). The findings demonstrated that LIPUS inhibited glial activation and the 6-hydroxydopamine-induced phosphorylation of NF-κB p65, thereby preserving the levels of neurotrophic factors, dopamine transporters, and tight junction proteins of the BBB.

Cognitive impairment is a common non-motor symptom in patients with PD, but current treatments show limited efficacy. In a study involving transgenic PD mice, the CA1 region of the hippocampus was stimulated with ultrasound for 7 days. The working memory ability of the mice was assessed using the novel object recognition test, while local field potentials and neuronal spikes in the CA1 region were recorded simultaneously during behavioral testing (Zhao et al., 2024). Ultrasound stimulation was found to enhance learning and memory abilities in PD mice, increasing the relative power of the theta, low gamma, and high gamma frequency bands in local field potentials. Additionally, this treatment modulated the phase-locking angle between interneuron spikes and theta waves. These findings suggest that neural oscillations in the CA1 region may be a potential mechanism through which ultrasound stimulation improves memory performance.

In models of MS, LIPUS has been shown to effectively reduce demyelination and promote remyelination. For example, a study by Yang et al. (2022) applied LIPUS treatment to demyelinated lesions in the hippocampus of rats. The results demonstrated that LIPUS significantly enhanced remyelination, increased the expression of myelin basic protein, and promoted the maturation of oligodendrocytes. Additionally, LIPUS reduced the activation of astrocytes and microglia, suppressed the neuroinflammatory response, and increased the expression of BDNF. These findings suggest that LIPUS can enhance remyelination by inhibiting glial cell activation, promoting BDNF release, and stimulating oligodendrocyte proliferation, ultimately leading to increased myelin basic protein (MBP) levels. The LIPUS-induced improvements in myelination in specific brain regions represent a promising therapeutic approach for treating myelin loss in MS.

#### Applications of low-intensity transcranial ultrasound in animal models

In a study involving rat models of VD, LIFUS was applied bilaterally to the medial prefrontal cortex over a 2-week period. The rats showed enhanced performance in working memory tasks, which was associated with improved synaptic function. LIFUS also increased cerebral blood flow and reduced neuroinflammation in the medial prefrontal cortex by inhibiting the Toll-like receptor 4 (TLR4)/NF-κB pathway and decreasing the levels of proinflammatory cytokines (Wang et al., 2022a). These findings suggest that LIFUS can improve working memory in VD rats by enhancing the neuronal environment and synaptic function, highlighting its potential as a noninvasive therapy for cognitive impairment related to CNS diseases

In a study by Laurene Abjean (2024), the effects of repeated LIFUS combined with MB were investigated in the TgF344-AD rat model of AD. The study aimed to assess the effect of a combination of LIFUS and MB on neuroinflammation and amyloid load. A single exposure to LIFUS combined with MB triggered a mild astrocyte and microglial response 24 hours after treatment. However, repeated LIFUS treatments resulted in microglial reprogramming and altered gene expression related to the inflammatory response, mitochondrial function, and energy metabolism. Although multiple exposures did not significantly modulate the levels of soluble or highly aggregated forms of Aβ (Aβ_40_ and Aβ_42_), the study demonstrated that the combination of LIFUS and MB could enhance the brain delivery of the anti-Aβ antibody Aducanumab. Thus, this combination may serve as a promising tool for improving drug delivery in the treatment of AD, potentially reducing the required dose of administered drugs and minimizing side effects. **Additional Table 3** provides details regarding the parameters and effects of LIPUS and LIFUS in recent animal experiments related to AD.

**Additional Table 3:** Summary of recent applications of LIPUS and LIFUS in AD animal models

Subject/Target	Ultrasound parameter	Result	Reference
C57BL/6J mice, 5xFAD AD model, hippocampus and cortex	FF = 1.0 MHz; ISPTA = 0.5 W/cm^2^; DC = 50%; SD = 1 min	Improved cognitive functions; reduced A3 deposition; inhibited NLRP3 inflammasome activation	He et al., 2024
C57BL/6J mice, vascular dementia model, bilateral temporal bones	FF = 0.5 MHz; AP = 1.3 MPa; DC = 5%	Improved cognitive functions; increased cerebral blood flow	Shindo et al., 2024
C57BL/6J mice, APP/PS1 AD model, hippocampus and cortex	FF = 0.5 MHz; AP = 0.34 MPa; DC = 5%; PRF = 500 Hz; SD = 1 s	Improved cognitive functions; reduced A3 deposition; inhibited NLRP3 inflammasome activation	Zhang et al., 2023
P301S tau transgenic mice, tauopathy model, right hemisphere	FF = 1.0 MHz; AP = 0.3 MPa; PRF = 1 Hz; SD = 120 s	No reduction in tau pathology; decreased microglial density	Géraudie et al., 2023
Wistar rats, Aβ_1-42_-induced AD model, hippocampus and cortex	FF = 3.3 MHz; I = 0.8 W/cm^2^; DC = 50%; PRF = 100 Hz	Improved cognitive functions; increased BDNF and NGF-3 levels	Tramontin et al., 2021
C57BL/6J mice, LPS-induced neuroinflammation and memory impairment model, hippocampus and cortex	FF = 1.0 MHz; ISPTA = 0.528 W/cm^2^; DC = 5%; PRF = 1 Hz; SD = 15 min	Improved cognitive functions; reduced Aβ deposition; inhibited TLR4/NF-kB signaling	Chen et al., 2019b
C57BL/6J mice, 5xFAD AD model, whole brain	FF = 1.875 MHz; PRF = 6.0 kHz	Improved cognitive functions; reduced Aβ deposition; increased cerebral blood flow	Eguchi et al., 2018
Wistar rats, aluminum-induced cerebral damage model, hippocampus and cortex	FF = 1.0 MHz; ISPTA = 528 mW/cm^2^; DC = 5%; PRF = 1 Hz; SD = 5 min	Improved cognitive functions; reduced Aβ deposition; Increased brain-derived neurotrophic factor, glial cell-derived neurotrophic factor, and vascular endothelial growth factor levels	Lin et al., 2015

The table indicates the potential of both LIPUS and LIFUS to improve cognitive functions, reduce Aβ deposition, and modulate neuroinflammatory responses in AD models, offering valuable insights for further research and clinical translation. AD: Alzheimer's disease; AP: acoustic pressure; Aβ: amyloid-beta; BDNF: brain-derived neurotrophic factor; DC: duty cycle; FF: fundamental frequency; I: intensity; ISPPA: spatial-peak pulse average; ISPTA: spatial-peak temporal average; LIFUS: low-intensity focused ultrasound stimulation; LIPUS: low-intensity pulsed ultrasound stimulation; NGF-β: nerve growth factor beta; NLRP3: NLR family pyrin domain containing 3; PRF: pulsed repetition frequency; SD: stimulation duration; TLR4/NF-kB: Toll-like receptor 4/nuclear factor kappa-light-chain-enhancer of activated B cells.

The BBB, which limits the delivery of drugs and gene therapy vectors, poses a major challenge in the treatment of NDDs such as PD. LIFU combined with MB technology holds promise for safely and effectively opening the BBB for targeted therapy. In nonhuman primates and PD patients, LIFU was used to open the BBB and deliver adeno-associated virus type 9 (AAV9) vectors. The effects of BBB opening and vector delivery were assessed using magnetic resonance imaging (MRI) and positron emission tomography (PET) imaging (Blesa et al., 2023). LIFU successfully opened the BBB in both monkeys and patients, enabling targeted delivery of AAV vectors without adverse effects. This approach is a novel and promising pathway for gene therapy in NDDs such as PD.

Curcumin has demonstrated potential for treating CNS diseases, but its low solubility and poor bioavailability across the BBB limit its applications. The combination of Cur-NBs with LIFU may address these limitations. Curcumin solubility was enhanced through melt-crystallization methods, and Cur-NBs were fabricated by encapsulating curcumin into lipid-poly(lactic-co-glycolic) acid (PLGA) nanobubbles. LIFU was then used to open the BBB and deliver curcumin to the deep brain of PD mice, followed by evaluation of its therapeutic efficacy (Yan et al., 2021c). **Additional Table 4** details the parameters and effects of LIPUS and LIFUS in recent PD animal experiments.

**Additional Table 4:** Summary of recent applications of LIPUS and LIFUS in animal models of PD

Subject/Target	Ultrasound parameter	Result	Reference
C57BL/6 mice, MPTP-induced PD model-CAl region of the hippocampus	FF = 1 MHz; DC = 5%; PRF = 1 Hz	Improved memory performance in PD mice; increased relative power in theta, low gamma, and high gamma frequency bands; enhanced phase-amplitude coupling	Zhao et al., 2024
Nonhuman primates (adult macaque monkeys) -putamen, substantia nigra	FF = 0.22 MHz; I = 1000 mW; microbubble infusion rate: 0.02 mL/s; microbubble infusion start time: 3-4 min	Successfully opened the BBB; achieved adeno-associated virus vector delivery; improved PD symptoms	Blesa et al., 2023
C57BL/6 mice, MPTP-induced PD model, Ml (primary motor cortex)	FF = 0.8 MHz; ISPTA = 76 mW/cm^2^; DC = 10%; PRF = 100 Hz; SD =1 ms	Regulated microglia activation; increased neurotrophic factor; reduced oxidative stress; promoted nerve repair and regeneration; improved motor symptoms	Zhong et al., 2023
Sprague-Dawley rats, 6-OHDA-induced PD model, substantia nigra pars compacta	FF=1 MHz; ISPTA = 528 mW/cm^2^; DC = 5%; PRF = 1 Hz	Inhibited 6-hydroxydopamine-induced glial activation; reduced phosphorylation of nuclear factor kB p65; preserved neurotrophic factor levels; stabilized BBB; increased dopamine transporter	Song et al., 2022
Sprague-Dawley rats, 6-OHDA-induced PD model, midbrain substantia nigra	FF = 1 MHz; ISPTA = 528 mW/cm^2^; DC = 5%; PRF =1 Hz	Increased tyrosine hydroxylase staining density; increased glial cell-derived neurotrophic factor protein levels; attenuated LCN2-induced neuroinflammation; improved motor function	Sung et al., 2022
C57BL/6 mice, MPTP-induced PD model-dopaminergic neurons	FF = 1 MHz; I = (123 ± 2.781) - (110.667 ± 3.138) mW/cm^2^; DC = 20%; PRF = 1000 Hz	Reduced central neurotoxicity; reduced loss of tyrosine hydroxylase-positive neurons in the dense part of the substantia nigra; decreased apoptosis in the substantia nigra region; alleviated motor and balance dysfunction	Chen et al., 2021
C57BL/6 mice, MPTP-induced PD model-striatum	FF = 1 MHz; DC = 5%; PRF = 1 Hz	Increased the solubility of curcumin; successfully delivered curcumin; improved PD symptoms	Yan et al., 2021c
MPTP-induced chronic PD mouse model-subthalamic nucleus	FF = 3.8 MHz; ISPTA = 430 mW/cm^2^; DC = 50%; PRF = 1000 Hz	Reduced activation of microglia and astrocytes; improved the latency time for the rotating rod test (*P* = 0.033) and reduced climbing time for the pole test *(P* = 0.016)	Zhou et al., 2021
C57BL/6 mice, MPTP-induced PD model-subthalamic nucleus	FF = 0.5 MHz; ISPTA = 255 mW/cm^2^; DC = 5%; PRF = 1000 Hz; SD = 50 ms	Increased dopamine levels in the substantia nigra-striatal pathway; promoted regeneration of dopaminergic neurons; improved motor function	Yuan et al., 2020
PC12 cells and MPTP-induced PD mouse model, dopaminergic cells	FF = 1 MHz; I = 0.1-0.3 W/cm^2^	Significantly increased (78.5%) DA content; restoration of DA content in the mouse model to 81.07% of control levels; return to normal levels of motor activity	Xu et al., 2020
MPTP-induced PD mouse model, subthalamic nucleus	FF = 3.8 MHz; ISPTA = 180 mW/cm^2^; DC = 50%; PRF = 1000 Hz; SD = 1 s	Protected DA neurons from MPTP neurotoxicity; Significantly increased latency time (*P* < 0.05) and decreased climb time (*P* < 0.05) during the rotating rod test after	Zhou et al., 2019
PC12 cells, MPP+-induced neurotoxicity	FF = 1 MHz; ISPTA = 30 or 50 mW/cm^2^; DC = 20%; PRF = 100 Hz	Activated K2P channels and stretch-activated ion channels; attenuated MPP+ induced neurotoxicity; reduced oxidative stress	Zhao et al., 2017

The table highlights the therapeutic potential of these techniques in improving motor symptoms, promoting neuroregeneration, and enhancing neuroprotective effects in PD models, laying a foundation for advancing treatments for PD. BBB: blood-brain barrier; DC: duty cycle; FF: fundamental frequency; FF: fundamental frequency; I: intensity; ISPPA: spatial-peak pulse average; ISPTA: spatial-peak temporal average; LIFUS: low-intensity focused ultrasound stimulation; LIPUS: low-intensity pulsed ultrasound stimulation; MPP+: l-methyl-4-phenylpyridinium; MPTP: 1-methyl-4-phenyl-1,2,3,6-tetrahydropyridine; PD: Parkinson's disease; PRF: pulsed repetition frequency; SD: stimulation duration.

Animal studies have demonstrated substantial advancements in neuroprotection, anti-inflammation, and neuroregeneration, laying the scientific groundwork for translating LIUS technology to human use (Song et al., 2022; Sung et al., 2022; Zhong et al., 2023). Tailoring LIUS parameters for specific brain targets and leveraging animal data will enhance the precision of future human trials, ensuring the validation of LIUS efficacy and safety.

### Clinical translation status and challenges

Ultrasonic neuromodulation technology has recently emerged as a focal topic in clinical research. The clinical translation of this technology in NDDs is actively progressing. By searching the ClinicalTrials.gov and WHO ICTRP platforms with the keywords “Low-intensity transcranial ultrasound” and “Neurodegenerative diseases,” as of February 2025, nine registered clinical trials related to AD and PD were identified. Among these, early-phase (phase I/II) trials accounted for 80%, and phase III trials have not yet been conducted on a large scale. Although four registered trials have been completed, their results have not been published. Additionally, five registered trials are ongoing or have not yet reported results (**Additional Table 5**).

**Additional Table 5:** Summary of recent clinical trials on the treatment of neurodegenerative diseases with LIPUS and LIFUS

Registration number	Clinical trial	Start and completion date
NCT06763692	The trial is a prospective, single-center, single-arm early feasibility study aimed at establishing the safety and tolerability of LIFUS for neuromodulation in patients with PD. The study is not yet recruiting and plans to enroll 15 participants. The primary outcome measure is the occurrence of treatment-emergent adverse events, while the secondary outcome measure is the effect of LIFUS on visuospatial function.	March 1, 2025/March 30, 2028
NCT04593875	The trial is a completed study that tested the feasibility of using LIPUS to treat motor symptoms in PD. The study involved 30 participants and assessed improvements in motor performance through various tests, including finger tapping, the 9-hole pegboard dexterity test, and the Unified Parkinson's Disease Rating Scale Section 3.	June 15, 2022/July 31, 2024
NCT06090292	The trial is a recruiting study that aims to understand the neuromodulatory effects of combining transcranial ultrasound stimulation and functional electrical stimulation on motor symptoms in PD patients. The study plans to enroll 15 participants and will assess motor evoked potential amplitude as the primary outcome measure.	June 13, 2023/June 13, 2025
NCT05997030	The trial is a recruiting study that aims to establish the safety and tolerability of LIFUS for neuromodulation in patients with mild cognitive impairment due to AD. The study plans to enroll 15 participants and will assess cognitive changes using the Alzheimer's Disease Assessment Scale-Cognitive Subscale.	October 12, 2023/September 30, 2025
NCT05417555	The trial is a recruiting study that aims to investigate whether LIPUS targeting a region of the brain involved in memory will affect brain activity and improve memory in people with mild cognitive impairment and mild AD. The study plans to enroll 144 participants and will assess changes in perfusion using arterial spin labeling functional magnetic resonance imaging signal and blood-oxygen-level-dependent functional connectivity.	September 1, 2022/July 31, 2026
NCT06158789	The trial is an active, nonrecruiting study that focused on comparing the efficacy of repetitive LIFUS-mediated BBB opening in AD with increased treatment sessions and shorter intervals. The study enrolled six participants and assessed changes in standard uptake value ratios on 8F-Florbetaben positron emission tomography, as well as scores from the Caregiver-Administrated Neuropsychiatry Inventory, the Korean Mini-Mental State Examination, and the Seoul Neuropsychological Screening Battery tests.	June 2, 2022/January 19, 2024
NCT06135051	The trial is a recruiting study that will evaluate a new form of noninvasive deep brain therapy (LIFUS) for individuals with AD. The study plans to enroll 40 participants and will assess cognitive function using the Montreal Cognitive Assessment, the Hamilton Depression Rating Scale, and amyloid PET imaging.	August 18, 2024/April, 2027
NCT03347084	The trial is a completed study that aimed to determine the feasibility of brief brain stimulation using LIFUS for individuals with mild cognitive impairment or mild AD. The study enrolled two participants and assessed changes in functional magnetic resonance imaging brain scans.	November 10, 2018/October 1, 2023
NCT03119961	The trial is a completed study that investigated the use of LIFUS associated with microbubble injection to open the BBB in AD. The primary outcome measures are changes in Florbetapir standard uptake value ratios and fluorodeoxyglucose metabolic uptake values in the region of interest for BBB opening.	June 26, 2017/October 7, 2020

The table provides an overview of recent clinical trials evaluating the therapeutic potential of LIPUS and LIFUS in neurodegenerativo diseases, particularly PD and AD. Most trials are in the early phases (Phase I/II), with small sample sizes and limited long-term data. The table highlights the need for further research to optimize neuromodulation parameters, validate therapeutic effects, and establish standardized protocols for broader clinical application. AD: Alzheimer's disease; BBB: blood-brain barrier; LIFUS: low-intensity focused ultrasound stimulation; LIPUS: low-intensity pulsed ultrasound stimulation; PD: Parkinson's disease.

The main challenges at present include the need for further optimization of neuromodulation parameters and the development of standardized treatment protocols in studies of NDDs (such as PD and AD) that have been conducted using ultrasonic neuromodulation technology. This includes determining the optimal frequency, intensity, and target areas for modulation. Moreover, since the sample sizes in most registered studies are relatively small, the conclusions drawn from these studies need to be further validated through multi-center, large-scale trials to confirm their reliability and generalizability.

More than 50 current ultrasonic neuromodulation-related projects are funded by the NIH, but none are directly targeted at NDDs. This indicates a certain research gap in this area, which requires more attention and investment.

Despite ongoing technological advancements in LIUS and the generation of many related technical patents, as a medical device technology, its clinical application requires strict regulatory approval processes, including Investigational Device Exemption or Premarket Approval. Currently, no LIUS devices have been approved for clinical treatment of NDDs such as PD and AD.

### Clinical applications

LIUS has been shown to exhibit a wide range of therapeutic effects in clinical research and has been successfully applied in the treatment of various neuropsychiatric disorders, including PD, AD, depression, disorders of consciousness, and neuropathic pain. Matt et al. (2024) reported that LIUS can potentially enhance cognitive function, emotional state, and motor abilities in patients, offering promising avenues for treating complex disorders.

#### Applications of low-intensity transcranial ultrasound in patients

LIPUS has shown therapeutic potential in patients with AD. Hiroaki Shimokawa et al. (2022) conducted a pilot study to evaluate the safety and efficacy of whole-brain LIPUS therapy in patients with early-stage AD. Their study included both an open trial and a randomized, double-blind, placebo-controlled trial. LIPUS therapy was administered bilaterally through the temporal bones for whole-brain irradiation, three times a week for 1 hour, under specific conditions (1.3 MPa, 32 cycles, 5% duty cycle). No adverse reactions were reported in the open trial (*n* = 5). In the randomized controlled trial (*n* = 22), the trend of worsening in the Japanese version of the Alzheimer’s Disease Assessment Scale–Cognitive Subscale (ADAS-Jcog) scores at 72 weeks was alleviated in the LIPUS group in comparison with the placebo group (*P* = 0.257). Defined as “responders,” patients who did not show worsening or even showed improved ADAS-Jcog scores at 72 weeks had a responder rate of 50% (5/10) in the LIPUS group, while the placebo group had a responder rate of 0% (0/5) (*P* = 0.053). These results suggest that LIPUS therapy is safe and may help suppress cognitive impairment in patients with early AD; however, larger-scale trials are needed to further verify these findings.

LIPUS has demonstrated remarkable ability to improve behavioral function in animal models. It non-invasively penetrates the skull and precisely modulates neural circuits in the CNS, resulting in positive therapeutic changes (Zhong et al., 2024). As shown by Grippe et al. (2024), LIPUS possesses a high degree of spatial specificity and can increase the excitability of the motor cortex (M1), similar to the phenomenon of long-term potentiation. Zhong et al. (2024) conducted the SWUMP trial to further validate the efficacy of LIPUS in a clinical setting. This rigorously designed, single-center, prospective, double-blinded, randomized controlled trial included 48 patients with clinically confirmed PD. In the trial, patients were randomized into a LIPUS group and a control group, with medication maintained as baseline therapy throughout. The LIPUS group received LIPUS treatment with specific parameters, while the control group received simulated treatments. The study aimed to evaluate the impact of LIPUS on motor function, quantified by changes in Movement Disorder Society (MDS)-unified Parkinson’s disease rating scale (UPDRS)-III scores, highlighting its therapeutic potential and clinical applicability.

In another case-control study by Grippe et al. (2024), the therapeutic efficacy of LIPUS in patients with moderate PD was explored in depth. The study included 20 patients with PD (7 with dyskinesia and 13 without) and 17 age-matched healthy controls. During the study, motor-evoked potentials (MEPs), short-interval cortical inhibition, and short-interval cortical facilitation indicators were recorded in patients with PD during the dopaminergic drug-switched state. The results showed that transcranial ultrasound stimulation elevated MEP amplitudes in both healthy controls and medicated PD patients after 30 minutes, with no significant effect observed in unmedicated PD patients. Stimulation also improved the motor retardation subscores in medicated PD patients, suggesting expanded clinical potential for LIPUS in PD treatment.

Additionally, LIPUS has demonstrated potential in treating neurological disorders, with Lee et al. (2021) notably implementing LIPUS in patients with drug-resistant epilepsy. Their treatment protocol led to a reduced seizure frequency in two cases, indicating novel therapeutic avenues for epilepsy management.

#### Applications of low-intensity transcranial ultrasound in patients

The therapeutic effects of LIFUS have also been explored in patients with AD. Jeong et al. (2021) assessed the effects of LIFUS on the hippocampal region of patients with AD. Their study included eight patients who received LIFUS treatment with specific parameters (250 kHz; pulse duration, 20 milliseconds; PRF, 2 Hz; and treatment duration, 180 seconds). The results showed that LIFUS significantly improved immediate recall (*P* = 0.03) and recognition memory (*P* = 0.02) in the verbal learning test. Additionally, PET analysis revealed a significant increase in the glucose metabolism rate (rCMRglu) in the right hippocampal region (*P* = 0.001), which correlated with the improvement in recognition memory (Spearman’s *ρ* = 0.77, *P* = 0.02). No adverse events were reported, indicating that LIFUS is a safe and effective method for enhancing cognitive function in AD patients.

Another study by Mahoney et al. (2023) evaluated the long-term safety and cognitive outcomes of LIFUS-mediated BBB opening in patients with mild AD. The study involved 10 patients who received three LIFUS treatments targeting the hippocampus, frontal, and parietal regions. The results showed that the BBB opened immediately after LIFUS in all patients and closed within 48 h. Cognitive assessments using the Mini-Mental State Examination and Alzheimer’s Disease Assessment Scale–Cognitive Subscale indicated stable cognitive function at 6 months and a decline at 1 year, similar to the Alzheimer’s Disease Neuroimaging Initiative cohort. PET scans revealed an average reduction of 5% in Aβ plaques in the regions treated with focused ultrasound, corresponding to a 14% reduction on the Centiloid scale. These findings suggest that LIFUS-mediated BBB opening is safe and may reduce Aβ plaques without accelerating cognitive decline in patients with AD. **Additional Table 6** provides details on the parameters and effects of LIPUS and LIFUS in the treatment of patients with AD patients in recent studies.

**Additional Table 6:** Summary of recent applications of LIPUS and LIFUS treatments for AD patients

Target	Ultrasound parameter	Result	Reference
Right hippocampus	FF = 0.25 MHz; AP = 0.2 Pa; PRF = 2 Hz	Successful BBB opening was achieved in 4 out of 5 patients; PET scans showed increased glucose metabolism; cognitive function tests showed improvement in memory functions.	Bae et al., 2024
Right hippocampus, frontal lobe, parietal lobe, and entorhinal cortex	FF = 0.22 MHz; AP = 0.5-1 MPa	MRI showed contrast enhancement, indicating BBB opening; PET scans showed a reduction in Aβ plaques.	Mehta et al., 2021; Mehta et al., 2023
Left supramarginal gyrus	FF = 1 MHz; AP = 0.9-1.03 Pa	Successful BBB opening was achieved. PET scans revealed a reduction in Aβ plaques; however, cognitive function tests did not demonstrate significant improvement.	Epelbaum et al., 2022
Bilateral temporal bones	FF = 0.5 MHz; AP = 1.3 Pa; DC = 5%; PRF = 781 Hz	Cognitive function tests indicated a slowing of cognitive decline.	Shimokawa et al., 2022
Right hippocampus	FF = 0.25 MHz; ISPTA = 0.5-3 W/cm^2^; DC = 4%; PRF = 2 Hz; SD = 20 ms	Improved memory and executive function; increased regional cerebral metabolic rate of glucose; considered safe and may enhance cognitive function.	Jeong et al., 2021, 2022

The table highlights the potential of these techniques to improve cognitive functions, enhance neuroplasticity, and modulate neuroinflammatory responses in AD patients. While LIPUS offers a noninvasive and portable option for widespread application, its effectiveness in deep brain modulation may be limited compared with that of LIFUS. LIFUS is distinguished by its precise acoustic focusing and its ability to temporarily open the BBB, thereby enhancing drug delivery and treatment efficacy. However, the complexity of LIFUS technology and the need for further research on its long-term safety and efficacy are important considerations. Future studies should focus on optimizing treatment protocols and validating their therapeutic effects to establish standardized clinical applications for AD patients. AD: Alzheimer's disease; AP: acoustic pressure; Aβ: amyloid-beta; BBB: blood-brain barrier; DC: duty cycle; FF: fundamental frequency; I: intensity; ISPPA: spatial-peak pulse average; ISPTA: spatial-peak temporal average; LIFUS: low-intensity focused ultrasound stimulation; LIPUS: low-intensity pulsed ultrasound stimulation; MRI: magnetic resonance imaging; PET: positron emission tomography; PRF: pulsed repetition frequency; SD: stimulation duration.

In a study involving patients with PD, LIFUS demonstrated that transcranial ultrasound stimulation of the motor cortex could effectively enhance the amplitude of MEPs and lead to improvements in bradykinesia symptoms (Grippe et al., 2024).

To further validate the efficacy of LIFUS in the clinical management of PD, Samuel et al. (2023) investigated accelerated transcranial ultrasound stimulation in 10 patients with PD over two sessions; the patients received either active treatment or sham stimulation spaced 10 days apart. The active accelerated transcranial ultrasound stimulation significantly elevated MEP amplitudes in comparison with sham treatment; however, no significant clinical differences were observed in the MDS-UPDRS-III score.

In a subsequent clinical trial involving five patients with PD treated with magnetic resonance-guided focused ultrasound targeting the right parieto-occipito-temporal cortex, which is key in Parkinson’s disease dementia (PDD), the findings demonstrated the safety and reversible effects of magnetic resonance-guided focused ultrasound. After treatment, patients exhibited minor cognitive improvements; however, PET scans for amyloid and fluorodeoxyglucose showed no significant alterations (Gasca-Salas et al., 2021).

Additionally, LIFUS technology has demonstrated promising capabilities in treating various neurological and psychiatric conditions. LIFUS can effectively induce potentiation in the primary visual cortex and enhance blood oxygen level-dependent signaling in functionally related brain regions, highlighting its potential to influence brain activity (Lee et al., 2015). It has also shown benefits in managing neuropathic pain, potentially improving mood and alleviating pain in chronic conditions (Hameroff et al., 2013). In the treatment of disorders of consciousness, LIFUS stimulation of the thalamus has been found to help awaken patients (Monti et al., 2016).

In the context of depression treatment, the positive effects of LIFUS on mood have been demonstrated, particularly when targeting the right frontotemporal cortex (Reznik et al., 2020; Sanguinetti et al., 2020). For AD, the combination of LIFUS and microbubble technology can open the BBB, improve drug-delivery efficiency, reduce Aβ plaques and tau protein cleavage, and restore neuronal function (El Khoury et al., 2010; Burgess et al., 2014). **Additional Table 7** provides details regarding the parameters and effects of LIPUS and LIFUS in the treatment of patients with PD.

**Additional Table 7:** Summary of recent applications of LIPUS and LIFUS for the treatment of PD patients

Target	Ultrasound parameter	Result	Reference
Dopaminergic neurons, motor cortex	FF = 0.6 MHz; I = 1 W/cm^2^; DC = 50%	Alleviated movement disorders	Zhong et al., 2024
Bilateral primary motor cortex (M1)	FF = 0.5 MHz; ISPTA = 290 mW/cm^2^; DC = 10%; PRF = 5 Hz	Induced long-lasting enhancement-like plasticity and alleviated motor retardation in the drugged state	Grippe et al., 2024
Bilateral primary motor cortex (M1)	FF = 0.5 MHz; ISPTA = 230 mW/cm^2^; DC = 10%; PRF = 5 Hz; SD = 200 ms	Enhanced amplitude of movement-related cortical potentials	Samuel et al., 2023
Right parieto-occipito-temporal cortex (right parieto-occipito-temporal junction)	Microbubble intravenous injection (i.v); acoustic energy up to 17.4 W; the average number of sound waves is 5.8	BBB opening, mild improvement in cognitive tests	Gasca-Salas et al., 2021

The table provides a summary of recent clinical trials evaluating the therapeutic potential of LIPUS and LIFUS in patients with PD. Each modality has unique advantages and limitations. LIPUS is noninvasive, portable, and relatively simple to use, making it widely applicable. It can promote neurite growth and neurotrophic factor expression, thereby enhancing motor and cognitive functions. However, LIPUS may be less effective than LIFUS in modulating deep brain structures and can be affected by the heterogeneity of the skull. In contrast, LIFUS offers precise acoustic focusing and effective modulation of deep brain structures. It can accurately target affected neurons, thereby enhancing treatment efficacy, and has the ability to temporarily open the BBB to improve drug and gene delivery. Nevertheless, LIFUS is complex, has potential side effects, and lacks long-term safety data. Future research should focus on optimizing treatment parameters, confirming long-term safety, and exploring personalized applications for different PD patients to develop more effective and safer therapies. BBB: Blood-brain barrier; DC: duty cycle; FF: fundamental frequency; I: intensity; i.v.: intravenous; ISPTA: spatial-peak temporal average; LIFUS: low-intensity focused ultrasound stimulation; LIPUS: low-intensity pulsed ultrasound stimulation; PD: Parkinson's disease; SD: stimulation duration.

No established methods are currently available for detecting neuronal regeneration in humans, and evaluations of treatment effects primarily rely on behavioral assessments. However, advancements in gene expression analysis and behavioral assessments hold promise for future breakthroughs. LIUS shows clinical potential for treating neuropsychiatric conditions but faces several challenges, including limited sample sizes, a lack of standardized protocols, insufficient long-term efficacy and side effect data, and heterogeneous treatment effects. Rigorous research is urgently required to refine these ultrasound methods and validate their safety and efficacy.

#### Safety

The existing concerns regarding transcranial focused ultrasound stimulation (tFUS) primarily revolve around the potential risks associated with applying focused ultrasound to the human brain. These risks include tissue damage due to increased tissue temperature, deformation of tissues as a result of ultrasound pressure, and damage caused by the rapid explosion of microbubbles within the cranial cavity. Ultrasound is a mechanical wave that, during propagation, is absorbed by tissues, leading to changes in tissue temperature. However, these changes are usually minimal because the skull significantly absorbs sound waves during transmission. Consequently, the sound energy that passes through the skull typically weakens to 20.9%–24.0% of the original intensity (Lee et al., 2021). Additionally, blood flow perfusion and biological heat exchange in tissue areas contribute to heat loss (Kiyatkin, 2019). Therefore, while ultrasound stimulation may cause significant temperature changes in the skull, the temperature change in brain tissue is generally minimal. Although the US Food and Drug Administration (FDA) and the International Electrotechnical Commission (IEC) recommend that the ISPTA should not exceed 3 W/cm^2^, calculations using a more precise bioheat equation indicate that, when ultrasound with an ISPTA of 7 W/cm^2^ passes through 3 mm of the skull, the increase in brain tissue temperature typically does not exceed 0.2°C, while the skull temperature increases by 2.8°C (Verhagen et al., 2019). Such temperature changes do not generally cause damage to brain tissue. Experimental results also indicate that the temperature change caused by neurostimulation using LIUS is less than 1°C, and does not have any negative effects on brain tissue (Jeong et al., 2021; Mahoney et al., 2023; Wang et al., 2023a, b). LIUS (ISPPA < 100 W/cm^2^) has relatively small thermal effects, and typically does not cause substantial temperature changes in brain tissue (Wang et al., 2019a; Cain et al., 2021). Moreover, no thermal damage has been observed even when using ultrasound with intensities greater than 100 W/cm^2^ for stimulation (Hameroff et al., 2013; Stern et al., 2021). The limited effect of temperature changes caused by the thermal effects of transcranial ultrasound stimulation on tissue has also been demonstrated in animal studies (Yoo et al., 2011; Chen et al., 2019a; Sharabi et al., 2019; Tsehay et al., 2023). Stimulation at an ISPPA of 27.2 W/cm^2^ in mice did not result in noticeable brain damage or behavioral abnormalities (Sharabi et al., 2019). Similarly, stimulation at an ISPPA of 18.2 W/cm² in 10 sheep showed no signs of thermal damage (Chen et al., 2019a). Using magnetic resonance (MR) thermometry, continuous stimulation at an ISPPA of 23 W/cm^2^ for 27 seconds in rabbits resulted in only a 0.7°C increase in temperature in the stimulated area (Yoo et al., 2011). In several studies reporting the thermal damage caused by transcranial ultrasound stimulation, ultrasound intensities significantly exceeded safety standards or the stimulation times were excessively long. For instance, stimulation with ultrasound at intensities greater than 60 W/cm^2^ for 30 seconds was applied to mice (Ballantine Jr et al., 1956). Additionally, stimulation at an intensity of 17 W/cm^2^ for 300 seconds was performed in rats (Oakden et al., 2014), and stimulation at intensities exceeding 200 W/cm^2^ for 1 second was used in cats (Lele, 1967), both of which resulted in spinal cord damage. However, this damage cannot be conclusively attributed to the thermal effects of ultrasound. Ultrasound may exert mechanical forces on cell membranes through acoustic pressure, altering membrane permeability or causing irreversible tissue deformation. These low-intensity mechanical effects are considered a key mechanism by which transcranial ultrasound influences neural activity (Nakajima et al., 2022). An important feature of LIUS is its ability to avoid excessive physical forces on brain tissue by precisely controlling sound wave pressure and mechanical effects. Studies have shown that micro-hemorrhage or ultrasound-induced damage does not occur when acoustic pressure is maintained below the threshold for BBB rupture (Daniels et al., 2018a, b, 2019; Chen et al., 2020; Jeong et al., 2021). Moreover, tFUS uses precise acoustic focusing technology to prevent transmission of excessive sound energy to non-target areas, thereby reducing the potential for damage (Kim et al., 2022). Some studies have suggested that ultrasound stimulation may have temporary effects on neural cells; however, no direct mechanical damage has been observed in human experimental reports (Hameroff et al., 2013; Hu et al., 2014; Lee et al., 2016a; Sarica et al., 2022).

In human experiments, ultrasound is typically applied at intensities below 2 MPa. Chronic pain patients exposed to an ultrasound intensity of 1.98 MPa reported no adverse reactions (Hameroff et al., 2013). In stroke models in mice, stimulation with an acoustic pressure of 3.7 MPa showed no evidence of tissue damage or bleeding in histological evaluations (Baek et al., 2018). Similarly, histological examination showed that ultrasound stimulation at 1.2 MPa in epileptic rats did not cause brain tissue damage, inflammation, or behavioral abnormalities (Chen et al., 2020). Although different ultrasound parameters were tested on eight sheep, microbleeding was observed in the primary visual cortex of four sheep after 600 stimulations with ultrasound at an ISPPA of 6.6 W/cm^2^. However, no abnormalities were found in the stimulated somatosensory-motor areas (Lee et al., 2016b), and the findings could not clearly determine whether the microbleeding was caused by mechanical stress.

The rupture of microbubbles can increase shear stress on surrounding cells and blood vessel walls, enhancing the permeability of cell membranes. While this effect is beneficial for drug or gene delivery, it can also lead to cell damage (Dijkmans et al., 2004). Although cavitation effects can cause localized damage at higher intensities, most of the existing studies have indicated that commonly used ultrasound intensities, particularly in cortical modulation, do not reach the threshold for cavitation. In fact, the occurrence of cavitation effects during LIUS applications is relatively rare (Blackmore et al., 2019; Reznik et al., 2020; Zhang et al., 2021b; Radjenovic et al., 2022). The MI is commonly used to assess the risk of cavitation effects, with the FDA setting the MI threshold at 1.9. Research by Mahoney et al. (2023) showed that at an MI of 2.72, participants experienced temporary scalp swelling. At an MI of 3.28, patients reported headaches and nausea, but all symptoms resolved quickly. Furthermore, using a moderate pulse duration can further reduce the likelihood of cavitation effects (Reznik et al., 2020). An experiment conducted on sheep demonstrated that a peak negative pressure of 12.7 MPa is required to induce bubble nucleation under a short-period pulse at 0.66 MHz (Gateau et al., 2011).

Mild adverse effects of tFUS are extremely rare, and no severe or persistent effects have been reported in human experiments. Furthermore, no adverse effects have been observed even in studies using stimulation parameters exceeding those recommended by the FDA (Pasquinelli et al., 2019). tFUS typically utilizes LIPUS, which helps to avoid rapid temperature increments, heat accumulation, and potential tissue damage caused by transient cavitation. However, no unified standard for safety parameters in transcranial ultrasound stimulation has been established at present, and the standards used in different studies vary significantly, affecting the comparability of results. Individual differences, such as skull thickness and density, may also lead to unstable stimulation effects or increase potential risks (Legon et al., 2014; Lee et al., 2016b; Monti et al., 2016). Moreover, the potential for chronic inflammation or activation of glial cells through long-term exposure to ultrasound remains to be verified through long-term animal studies. Despite the preliminary evidence regarding the safety and feasibility of tFUS, additional research is required to clarify its dose-response curve, the effects of repeated use, and the impact of variations in cranial anatomy.

## Limitations

The mechanisms of LIUS-based noninvasive neuromodulation have not been fully clarified. For example, further research is required to understand how this modality regulates neuronal excitability, promotes the release of neurotrophic factors, and improves BBB permeability. In addition, the specific mechanisms underlying LIFUS-induced BBB modulation and neural stem cell activation remain to be determined.

The ultrasound parameters used in existing studies differ substantially, and the lack of a unified dose-effect model limits the comparability of research results and the evaluation of therapeutic effects (Gasca-Salas et al., 2021; Samuel et al., 2023; Grippe et al., 2024; Zhong et al., 2024). For instance, the ultrasound frequency, acoustic intensity, PRF, and ultrasound duration used in different studies vary considerably, making direct comparisons difficult (Jeong et al., 2021, 2022; Epelbaum et al., 2022; Shimokawa et al., 2022; Bae et al., 2024).

LIUS shows great potential in treating NDD, but cranial heterogeneity poses a major challenge. Variations in skull thickness and density can weaken ultrasound energy, affecting the precise targeting of the treatment area. To tackle this, treatment plans must be optimized through individualized CT/MRI modeling. For instance, differences in skull thickness and density can lead to 30%–75% reduction in ultrasound energy, directly affecting treatment efficacy.

Clinical trials in this field are predominantly small-scale and single-center studies, limiting the availability of long-term efficacy data. This restriction hampers comprehensive assessments of the effectiveness and safety of the technology across various NDDs. For instance, two ongoing clinical trials of LIFUS for PD (NCT04250376 and NCT05475340) are single-center studies with limited sample sizes and no long-term follow-up data.

The therapeutic response to LIUS is influenced by multiple factors, such as age, sex, and the severity of the NDD. This complexity has necessitated the development of personalized treatment protocols. In PD, atrophy of the substantia nigra can reduce ultrasound energy absorption, necessitating parameter adjustments. Moreover, given individual differences, the efficacy and safety of LIPUS can vary across different diseases.

## Summary

### Technical advantages and safety assessment

Although the current basic and clinical research is still relatively limited, LIUS is emerging as an alternative to conventional neuromodulation, with the potential to enhance cognitive, emotional, and motor functions in patients, suggesting novel treatment options for complex diseases (Baek et al., 2017). Its noninvasiveness bypasses infection and surgical risks, whereas its high spatial resolution allows precise targeting of brain regions, supporting research into functional deficits (Tanimoto et al., 2008; Yoo et al., 2011). In addition, its deep penetration ability allows deeper brain structures to be within the target range and shows a wider range of applicability than traditional noninvasive brain stimulation techniques (Yoo et al., 2011). It can modulate neuroplasticity and motor cortex excitability, offering new strategies for improving NDD symptoms, and complement pharmacological treatments to increase neuroplasticity (Grippe et al., 2024). Furthermore, owing to its multimodal applications, LIUS serves not only as a therapeutic tool but also as a research instrument that enhances our understanding of brain functions and disease mechanisms (Eisenberg et al., 2021).

LIUS does not generate heat to damage the tissue and therefore avoids the risk of thermal injury (El Khoury et al., 2010). In animal models, LIUS also does not cause structural damage to brain tissue, which further demonstrates its safety (Burgess et al., 2014). Moreover, the stimulation effect can be precisely controlled by adjusting the parameters, which makes the treatment process safer and more controllable (Fomenko et al., 2020; Zhong et al., 2024). The results of clinical trials have shown that the technique has a good safety profile with mild and temporary side effects and causes no serious side effects or complications (Legon et al., 2020; Eisenberg et al., 2021). Safety is further ensured by the fact that the intensity of the sound waves complies with the U.S. Food and Drug Administration (FDA) guidelines for diagnostic ultrasound (Zhong et al., 2024). Long-term follow-up results have shown that the therapeutic effects of LIUS are sustainable over the long term with no delayed side effects (Cosgrove et al., 2022), and personalized treatment strategies further increase treatment safety and efficacy (Natera-Villalba et al., 2024).

Importantly, LIUS must exclude patients with elevated intracranial pressure to avoid complications from transcranial ultrasound (Robba et al., 2019), and its safety in those with intracranial metal implants, such as deep brain stimulators, requires evaluation (Legon and Strohman, 2024). In patients with a bleeding tendency, the risk of hemorrhage induced by transcranial ultrasound should be evaluated (Daffertshofer et al., 2005). Conducting comprehensive investigations to identify and characterize additional contraindications is essential for ensuring the safe application of LIUS.

### Difficulties and challenges

While LIUS has shown promise in treating NDDs, several challenges remain. Although LIUS can effectively alleviate NDD symptoms, the exact neurobiological mechanisms underlying its effects are still unclear (Wang et al., 2019b). Additionally, technical limitations and the need for precision present significant hurdles. Owing to anatomical variations among patients, ensuring accurate and consistent energy delivery to the target area during LIUS treatment is highly challenging (Eisenberg et al., 2021; Natera-Villalba et al., 2024). Overcoming this challenge is crucial for improving therapeutic outcomes and realizing the full potential of LIUS in clinical applications and research.

During LIUS treatment for NDDs, patients may detect auditory sounds, which allows them to distinguish between stimulated and nonstimulated trials. This auditory perception could influence experimental outcomes, as participants might infer whether they received LIUS on the basis of the sounds they heard.

Electroencephalogram recordings have indicated auditory activation associated with LIUS, suggesting potential indirect activation of early auditory pathways. To mitigate this auditory interference, trials have utilized earplugs or white noise to help most participants avoid distinguishing between real and sham stimulation. However, some individuals can still detect stimulation, possibly due to variations in skull resonance (Braun et al., 2020). In sham-controlled studies, actual treatment parameters are applied, but the ultrasound is dispersed to mimic a nonfocused state. The same analysis methods used for blood-oxygen-level-dependent fMRI data account for these auditory effects (Riis et al., 2024). Moreover, verifying the long-term efficacy and safety of LIUS presents another crucial challenge. While LIUS shows short-term promise, there are significant gaps in our understanding of its long-term benefits and safety, largely due to the lack of long-term follow-up studies and effective management of potential side effects (Gibson et al., 2018; Braun et al., 2020; Krishna et al., 2023). Additionally, the complexity of the equipment and procedures increases treatment costs, which limits access for a broader patient population (Lu et al., 2023).

### Prospects

Neuromodulation for NDDs is advancing through the precise targeting of neural networks and specific brain regions. Progressing this technology requires cross-disciplinary collaboration and may involve integrating brain imaging techniques such as fMRI and electroencephalography to monitor and adjust neural activity.

Personalized treatment is essential for improving therapeutic efficacy and safety. Imaging techniques such as diffusion tensor imaging, arterial spin labeling, and fMRI can reveal subtle pathological changes in NDDs, reflecting the neurodegenerative process (Lenfeldt et al., 2015; Pelizzari et al., 2019; Miao et al., 2022). Image-guided programming combines preoperative MRI with postoperative CT scans, utilizing commercial software such as GUIDE XT^TM^ to visualize DBS electrode positions and simulate the volume of tissue activated. This approach enables precise adjustments to stimulation locations and parameters in three-dimensional space, optimizing treatment (Waldthaler et al., 2021; Torres et al., 2024). In the future, integrating these imaging technologies and software algorithms could increase the precision of LIUS for NDDs, paving the way for more personalized and effective treatments.

**Figure 3:**
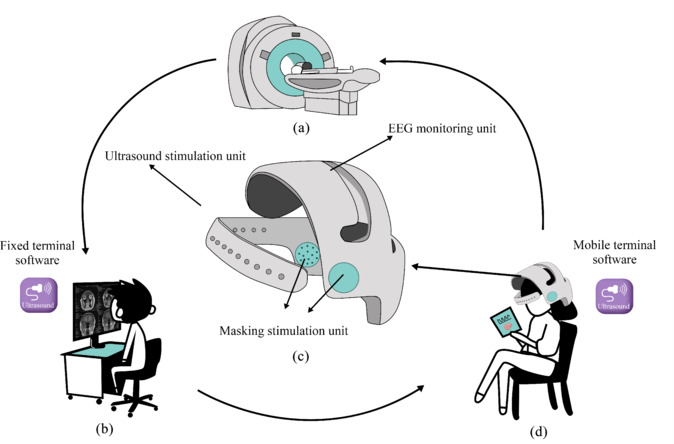
Envision process for future personalized treatment of NDDs. (a) Professional examination: Patients with NDDs undergo cranial imaging examinations at medical institutions to obtain detailed imaging data on brain structure and function. (b) Treatment planning: Medical staff, using imaging data and algorithmic analysis, create personalized treatment plans for patients and upload these plans to the “Ultrasound” Fixed Terminal Software. (c) Home treatment: Patients connect a wearable ultrasound neuromodulation device to the “Ultrasound” mobile terminal software at home for convenient ultrasound treatment. The software monitors progress and provides real-time feedback. (d) Fine tuning: The ultrasound stimulation unit precisely emits ultrasonic waves, whereas the masking stimulation unit reduces auditory interference. The EEG monitoring unit records changes in the electroencephalogram, synchronizing data with the software. If any abnormalities are detected, the software alerts patients to return to the hospital for re-examination and adjustment of their treatment plan. EEG: Electroencephalography; NDD: neurodegenerative disease.

LIUS technology is still in its early stages, and extensive animal studies are needed to validate its efficacy and safety. Future development must prioritize simplifying operations, reducing complexity, and enhancing portability and wearability (Zhong et al., 2024) to expand the application of LIUS in treating NDDs. Future human studies should prioritize addressing auditory interference, implementing masking techniques, and using advanced statistical methods to reduce nonspecific effects (**Figure 3**).

PD is a complex NDD, and its pathogenesis is linked to various cell death pathways, including apoptosis, necroptosis, and ferroptosis (Cui et al., 2025). Research in several areas, such as regulating BCL-2 family members, inhibiting the TNF and Fas signaling pathways, suppressing the activity of RIP1 and RIP3, modulating iron metabolism, increasing GPX4 activity, regulating CYPD activity, inhibiting excessive activation of PARP1, and enhancing TRIM31 function, is promising. These efforts aim to reduce neuronal death and damage while promoting cell regeneration and repair. New therapeutic targets and drugs, including PARP1 inhibitors, Withaferin A, and ferroptosis inhibitors, are continuously being researched and developed, offering potential breakthroughs in the treatment of PD and improving patients’ quality of life (Olsen and Feany, 2019; Abeesh and Guruvayoorappan, 2024; Xu et al., 2025b). Notably, studies indicate that LIUS may play a role in enhancing the proliferation and differentiation of stem cells (Min et al., 2022; Maimaitili et al., 2023; Chen et al., 2024b). LIUS has also been utilized to guide human pluripotent stem cells toward becoming dopaminergic neuron precursor cells, providing a new cellular resource for cell replacement therapy in NDDs (Maimaitili et al., 2023; Chen et al., 2024b). Additionally, LIUS stimulates the migration and differentiation of neural stem cells and promotes their integration into neural networks, supporting neural repair (Liang et al., 2021; You et al., 2023). As LIUS has emerged as a promising neuromodulation alternative, ongoing studies may uncover its full therapeutic potential, raising optimism for its future role in treatment.

## Additional files:

***Additional Table 1:*** *Summary of available neuromodulation techniques for the treatment of NDDs.*

***Additional Table 2:*** *Search strategy for the PubMed, Google Scholar, and Sci-Hub databases.*

***Additional Table 3:*** *Summary of recent applications of LIPUS and LIFUS in AD animal models.*

***Additional Table 4:*** *Summary of recent applications of LIPUS and LIFUS in animal models of PD.*

***Additional Table 5:*** *Summary of recent clinical trials on the treatment of neurodegenerative diseases with LIPUS and LIFUS.*

***Additional Table 6:*** *Summary of recent applications of LIPUS and LIFUS treatments for AD patients.*

***Additional Table 7:*** *Summary of recent applications of LIPUS and LIFUS for the treatment of PD patients.*

## Data Availability

*All relevant data are within the paper and its Additional files*.
